# Expression Pattern and Functional Characterization of *PISTILLATA* Ortholog Associated With the Formation of Petaloid Sepals in Double-Flower *Eriobotrya japonica* (Rosaceae)

**DOI:** 10.3389/fpls.2019.01685

**Published:** 2020-01-17

**Authors:** Yan Xia, Min Shi, Weiwei Chen, Ruoqian Hu, Danlong Jing, Di Wu, Shuming Wang, Qingfen Li, Honghong Deng, Qigao Guo, Guolu Liang

**Affiliations:** ^1^Key Laboratory of Horticulture Science for Southern Mountains Regions of Ministry of Education, College of Horticulture and Landscape Architecture, Southwest University, Chongqing, China; ^2^State Cultivation Base of Crop Stress Biology for Southern Mountainous Land of Southwest University, Academy of Agricultural Sciences of Southwest University, Chongqing, China; ^3^College of Forestry and Landscape Architecture, South China Agricultural University, Guangzhou, China

**Keywords:** *Eriobotrya japonica*, double-flower, *PISTILLATA*, MADS-box gene, expression pattern, ectopic expression

## Abstract

Double-flower *Eriobotrya japonica*, of which one phenotype is homeotic transformation of sepals into petals, is a new germplasm for revealing the molecular mechanisms underlying the floral organ transformation. Herein, we analyzed the sequence, expression pattern and functional characterization of *EjPI*, which encoded a B-class floral homeotic protein referred to as *PISTILLATA* ortholog, from genetically cognate single-flower and double-flower *E. japonica*. Phylogenetic analysis suggested that the *EjPI* gene was assigned to the rosids PI/GLO lineage. Analysis of protein sequence alignments showed that EjPI has typical domains of M, I, K, and C, and includes a distinctive PI motif at the C-terminal region. Compared with asterids PI/GLO lineage, the K1 and K3 subdomains of EjPI both contain a single amino acid difference. Subcellular localization of EjPI was determined to be in the nucleus. Expression pattern analysis revealed that *EjPI* expressed not only in petals, filament, and anther in single-flower *E. japonica*, but also in petaloid sepals in double-flower *E. japonica*. Meanwhile, there were high correlation between *EjPI* transcript level and petaloid area within a sepal. Furthermore, 35S::*EjPI* transgenic wild-type Arabidopsis caused the homeotic transformation of the first whorl sepals into petaloid sepals. Ectopic expression of *EjPI* in transgenic *pi-1* mutant Arabidopsis rescued normal petals and stamens. These results suggest expression pattern of *EjPI* is associated with the formation of petaloid sepal. Our study provides the potential application of *EjPI* for biotechnical engineering to create petaloid sepals or regulate floral organ identity in angiosperms.

## Introduction

Structural diversification of flower organs has often been considered to be function requirements of floral pollination/ecology biology (Endress, 2006). Understanding the underlying mechanisms for the diversification of floral organs has long been a challenge in angiosperms (Schwarz-Sommer et al., 1990; Frohlich, 2006; Irish, 2010). However, the most well-known ABCE-model maintains that each whorl organ identity is determined in a combinational way of four class homeotic proteins, termed A, B, C, and E class proteins (Bowman et al., 1991; Coen and Meyerowitz, 1991; Theißen, 2001; Theissen and Saedler, 2001; Litt and Kramer, 2010). According to this model, sepal identity is specified by the combinational A and E class proteins; petal formation is regulated by the combination of A, B, and E class proteins; stamen identity is specified by the combinational B, C, and E class proteins; carpel formation is regulated by the combination of C and E class proteins. All the A, B, C, and E proteins, except for *APETALA2*, belong to MIKC-type MADS-box transcription factors (Irish, 2010).

The MIKC-type MADS-box transcription factors are identified originally as floral homeotic proteins, and exhibit the characteristic domains from N- to C-terminus: a MADS (M), an intervening (I), a keratin-like (K), and a C-terminal (C) domains (Purugganan et al., 1995; Theißen et al., 1996; Theissen et al., 2000; Kaufmann et al., 2005; Litt and Kramer, 2010). Among these domains, the M domain, which is the most highly conserved region, contributes to the dimerization and nuclear localization (Gramzow and Theissen, 2010). The I domain, a relatively weakly conserved region, is also important for the DNA-binding dimer formation (Kaufmann et al., 2005). By contrast, the relatively conserved K domain, which allows a coiled-coil secondary structure by the formation of three-segment amphipathic helices, is involved in the formation of multimeric complex and protein dimerization (Yang and Jack, 2004; Puranik et al., 2014). Finally, the C domain, which is quite variable, contributes to the transcriptional activation and the formation of multimeric complex (Kaufmann et al., 2005; Theißen and Gramzow, 2016). Sequence differences of the MADS-box proteins from different flowering plant species has been used to clarify the evolution and diversification of floral organ identity (Theißen et al., 2016).

*PISTILLATA* (*PI*) homologues, which encode floral homeotic B-function MADS-box transcription factors, play a crucial role in the specification of petal and stamen identities in angiosperms (Goto and Meyerowitz, 1994; Hernandez-Hernandez et al., 2007; Whipple et al., 2007; Irish, 2009; Theißen et al., 2016). Molecular evolution of *PI* lineages indicates that the *PI* homologues are generated from a major duplication event of an ancestral gene, and their encoding proteins include a highly conserved PI motif in most angiosperms (Kramer et al., 1998). Therefore, functional diversification or conservation of *PI* orthologous genes need to be focused after the duplication events in different clades of angiosperms. The altered expressional patterns of *PI* orthologs in some angiosperm shaped floral organ diversification (Soltis et al., 2007; Viaene et al., 2009; Chen et al., 2012; Hofer et al., 2012; Sasaki et al., 2014; Jing et al., 2015; Dodsworth, 2017). However, the expression pattern and functional roles of *PI* ortholog in *Eriobotrya* remain unclear.

Double flower is one of the earliest documented examples of floral mutants (Meyerowitz et al., 1989). In many land plants, double-flower cultivars are selected as ornamentals and provide resources for elucidating the genetic difference between normal and double-flower phenotypes (Dubois et al., 2010; Galimba et al., 2012). Compared with the single-flower phenotype in angiosperms, extra petals of double flower are from homeotic transformation of the first whorl sepals or the third whorl stamens. At present, the formation of double flower in few species has been reported and mainly focus on the transformation of stamens into petaloid organ. Recent studies indicated that the transformation from stamen to petal in double flowers is associated with expression patterns of C-class genes (Dubois et al., 2010; Galimba et al., 2012; Liu et al., 2013; Ma et al., 2018). However, the regulatory mechanisms underlying the transformation from sepals to petals need further research.

**Figure 1:**
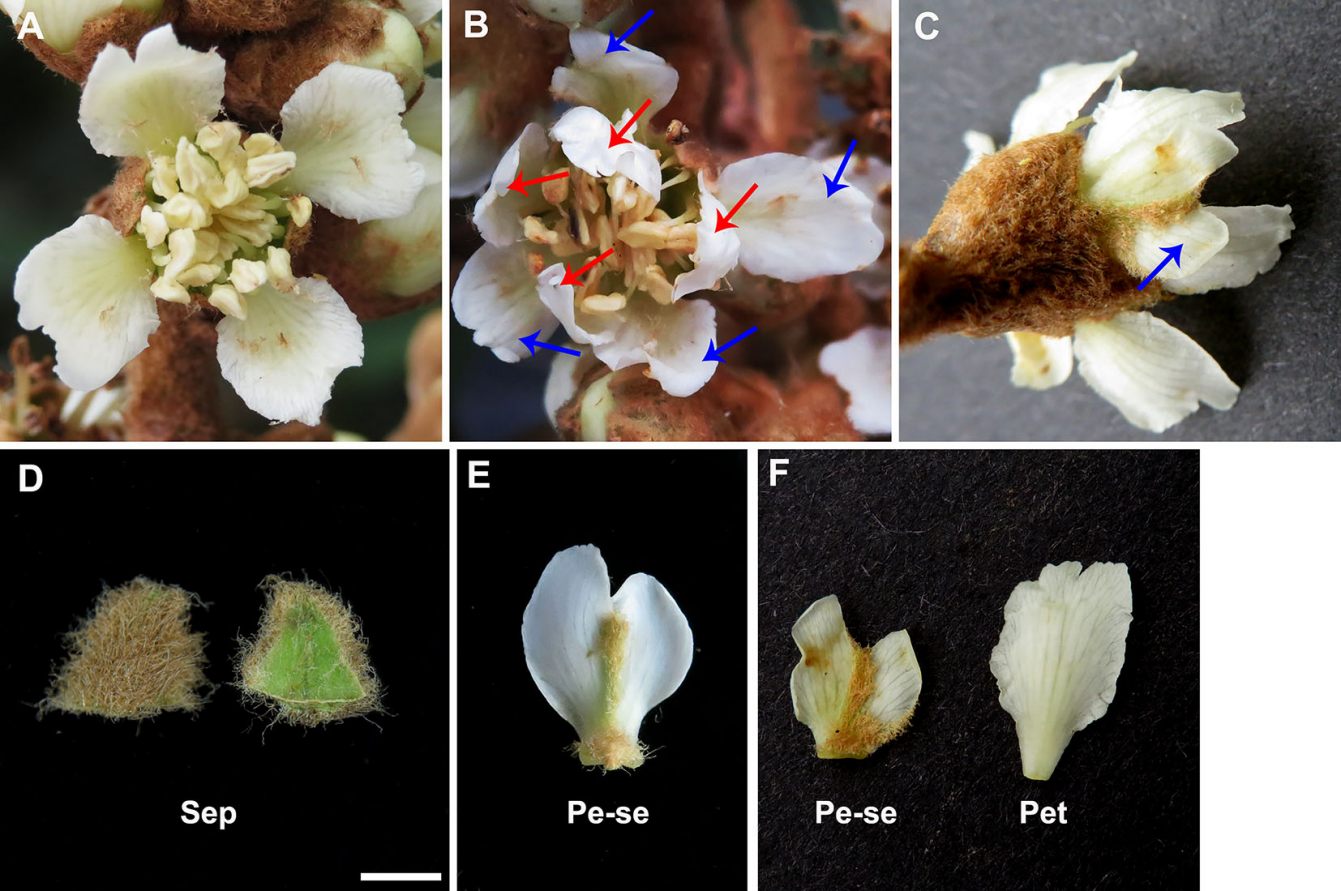
Comparative morphological observation in the single-flower and double-flower of *E. japonica*. **(A)** Single-flower *E. japonica*; **(B)** Double-flower *E. japonica*, showing homeotic conversional petaloid sepals from sepals in the first whorl (blue arrows) and petals in the second whorl (red arrows). **(C)** Petaloid sepals (blue arrow) in double-flower *E. japonica*. **(D)** Sepal. **(E)** Petaloid sepal. **(F)** Comparison of petaloid sepal and petal. Sep, sepal; Pe-se, petaloid sepals; Pet, petal.

*Eriobotrya japonica*, a Chinese originated evergreen tree, belongs to the family Rosaceae and is cultivated broadly in tropical and subtropical regions (Lin et al., 1999). The single-flower *E. japonica* has four normal floral whorls, which include four sepals in the first whorl, four petals in the second whorl, numerous stamens in the third whorl and five carpels in the fourth whorl (**Figures 1A, D**). However, the double-flower *E. japonica*, a recently discovered natural variation, has four floral whorls including four homeotic conversional petaloid organ from sepals in the first whorl, (**Figures 1B**–**F**), four petals in the second whorl, numerous stamens in the third whorl and five carpels in the fourth whorl. In this study, we isolated and identified *EjPI* gene, a *PI* ortholog, from genetically cognate single-flower and double-flower *E. japonica*. Analyses of protein sequence alignment and phylogenetic tree showed that *EjPI* is a typical B-class MADS-box genes and assigned to the rosids PI/GLO lineage. Expression pattern analysis suggested that *EjPI* expressed not only in petals, filament and anther in single-flower *E. japonica*, but also in petaloid sepals in double-flower *E. japonica*. Meanwhile, the expression level of *EjPI* was highly correlated with petaloid area within a sepal. The 35S::*EjPI* transgenic wild-type Arabidopsis caused the first whorl sepals replaced into petaloid sepals. Ectopic expression of the *EjPI* in homozygous *pi-1* mutant Arabidopsis rescued normal petals and stamens. These results reveal that expression pattern of *EjPI* is associated with the formation of petaloid sepal in double-flower *E. japonica*. Our study contributes to better understand the roles of *EjPI* for homeotic transformation of sepals into petaloid organs in double-flower *E. japonica*.

## Materials and Methods

### Plant Materials

At different development stages, flower buds from the single-flower and double-flower *E. japonica* were collected from an experimental farm of Southwest University (Chongqing, China). Sepals, petals, filaments, anthers, and carpels were sampled from single-flower *E. japonica*. The petaloid sepals, petals, filaments, anthers, and carpels from double-flower *E. japonica* were collected, and immediately frozen in liquid nitrogen. The seeds of *pi-1* mutant Arabidopsis (Landsberg *erecta*, CS77) were obtained from the Arabidopsis Biological Resource Center (Ohio State University, Columbus, OH, USA).

### Isolation of *EjPI* in *E. japonica*

Total RNA from floral buds of single-flower and double-flower *E. japonica* was extracted using EASYspin plant RNA Extraction kit (RN09, Aidlab, China). The 3′ rapid amplification of cDNA ends (RACE) of *EjPI* was conducted from the DNase I-treated RNA using a 3′-full RACE Core Set Version 2.0 kit (Takara, Japan). The primers of 3′ RACE, 3REjPI1, and 3REjPI2 were designed according to the conserved sequences of *PI* orthologs from related species in Rosaceae, such as *Kerria japonica* (GenBank accession number MH161203), *Pyrus pyrifolia* (KP164019), *Prunus pseudocerasus* (KM243373), and *Malus domestica* (AB081092). Then, first PCR was conducted using the gene-specific primer 3REjPI1. The nested PCR was conducted using the gene specific primer 3REjPI2. Furthermore, the primers of 5′ RACE, 5REjPI1, and 5REjPI2 were designed based on the obtained sequences of 3′ RACE. The 5′ partial cDNA of *EjPI* was isolated using the SMARTer RACE 5′/3′ kit (Takara, Japan). Then, first PCR was conducted using the gene specific primer 5REjPI1. The nested PCR was conducted using the gene specific primer 5REjPI2. To further verify the full-length cDNA sequence of *EjPI*, PCR was conducted using the primers of FLEjPIF and FLEjPIR. PCR parameters was at 94°C denaturation step for 5 min, followed by 35 cycles of 50 s at 94°C, 50 s annealing at 57°C and 50 s extension at 72°C, with a final extension period of 72°C for 10 min. These primers of PCR are shown in **Table S1**.

### Sequence Alignments and Phylogenetic Analysis

The BLAST analysis of deduced amino acid sequences of EjPI was performed on the Genbank database. Multiple A, B, C, and E class proteins were selected for alignment from various angiosperm lineages. Accession numbers of these proteins are listed in the **Table S2**. Amino acid sequences of these proteins, which contain the M, I, K, and C domains, were aligned using a ClustalW program (Thompson et al., 1994). A phylogenetic tree was constructed using MEGA 5.0 software (Kumar et al., 2008; Tamura et al., 2011) and the method described by Jing et al. (2015). Parameters of the phylogenetic tree were the bootstrap of 1,000 replicates, substitution model of Jones–Taylor–Thornton (Jones et al., 1992), uniform rates, nearest neighbor interchange, and the complete deletion of gaps/missing data. Lower than 50% of the values at each node was hidden.

### Subcellular Localization, Semi-Quantitative Reverse Transcription PCR and Quantitative Real-Time PCR (qRT-PCR)

Subcellular localization of *EjPI* was detected using the modified pCAMBIA 1300 vector (Liu et al., 2017) and *Agrobacterium*-mediated transient transformation in *Nicotiana benthamiana* leaves. Then, fluorescence signals of green fluorescent protein (GFP) were observed *via* fluorescent microscopy using an Observer DP80 (Olympus, Japan). As a control, the vector expressing GFP alone was used.

Total RNA concentrations of sepals, petaloid sepals, petals, filament, anther, and carpels were assayed using a Nanodrop 2000 Spectrophotometer (Thermo Scientific, USA). To synthesize first-strand cDNA, 2 µg of the DNase I-treated RNA was used with oligo (dT)-18 adaptor primer and M-MLV reverse transcriptase (Takara, Japan). Then, semi-quantitative RT-PCR was conducted using 2 µl of the cDNA, the forward primer of RTEjPIF and the reverse primer of RTEjPIR. The PCR products from each reaction were determined using a 1% agarose gel electrophoresis. The experiments were assayed for three independent biological replicates for each sample. As an internal control, the *ACTIN* gene of *E. japonica* was used with the specific primers RTEjactinF and RTEjactinR (Shan et al., 2008). These primers of semi-quantitative PCR are shown in **Table S3**.

Flower organ samples (200 mg) were collected, respectively from sepals, petals, and different petaloid sepals, i.e., ~34%, ~45%, ~65%, and ~86% petaloid area within a sepal. Three independent biological replicates were collected for each sample. Then, total RNA from sepals, petaloid sepals and petals, was extracted individually and treated with RNase-free DNase I (Takara, Japan). To generate the first-strand cDNA, 2 µg of total RNA were used using PrimeScript RT reagent Kit with gDNA Eraser (Takara, Japan). Then, cDNA was added in a 20 µL PCR reaction with the primers of QEjPIF and QEjPIR. The qRT-PCR were assayed using the SYBR green I (Takara, Japan) and CFX96 Touch Real-time PCR Detection System (Bio-Rad, USA). The reaction mixture was cycled using the previous parameters described by Jing et al. (2015). As an internal control, the *E. japonica β-actin* was used to normalize small differences in template amounts with the primers qEjactinF and qEjactinR (Shan et al., 2008). These primers of qRT-PCR are shown in **Table S3**. Three biological replicates for each sample were conducted. The relative quantification of gene expression level was determined by the 2^-ΔΔCT^ method (Livak and Schmittgen, 2001).

### Vectors Construction and Arabidopsis Transformation

Coding sequences of *EjPI* was cloned into a pBI121 vector (BD Biosciences Clontech, USA) using restriction enzymes of *Xba*I and *Sma*I (Takara, Japan). The 35S::*EjPI* construct was transformed into heterozygous *pi-1* mutant Arabidopsis lines *via* the *Agrobacterium tumefaciens* strain GV3101-90 using the floral-dip method described by Clough and Bent (1998). The seeds of transgenic Arabidopsis lines were selected using the previous method described by Jing et al. (2015). Then, the seedlings were transplanted in soil. The 35S::*EjPI* transgenic lines were detected by PCR and qRT-PCR. Genotype of transgenic Arabidopsis lines were identified using the primers of PI-1MF and PI-1MR designed by the dCAPS Finder program (Neff et al., 2002; Lamb and Irish, 2003; Jing et al., 2015). After genotyping, transgenic Arabidopsis lines of wild-type and homozygous *pi-1* mutant were observed. For qRT-PCR analysis of transgenic Arabidopsis lines, three biological replicates were performed using the primers of QEjPIF and QEjPIR, qApiF and qApiR. The *Actin* gene of Arabidopsis was used to normalize small differences in template amounts with the primers of qAactinF and qAactinR (Zhang et al., 2014). The primers of these PCR are shown in **Table S3**.

### Analysis of Scanning Electron Microscopy

Epidermal cells of floral organ from different Arabidopsis lines were fixed in 2.5% glutaraldehyde solution at 4°C for 48 h. The materials were dehydrated in a graded ethanol series and introduced at a critical point into the liquid CO_2_. The dried samples were coated with gold-palladium using a Hitachi E-1010 sputter Coater (Hitachi, Japan). Epidermal cells of the samples were observed using a FEI-Quanta 200F scanning electron microscope (FEI Company, Hillsboro, USA).

## Results

### Isolation and Sequence Analyses of *EjPI*

To isolate the *EjPI* sequence, we performed RACE technique to obtain full-length cDNA of *EjPI* from *E. japonica* flower bud (**Figure S1**). The *EjPI* cDNA sequence was 988 base pairs (bp) including a 61-bp of 5′ untranslated region (UTR), 648-bp open reading frame and 279-bp 3′ UTR with a poly-A tail at 3′-end (**Figure S2**). Isoelectric points and molecular weight of EjPI protein is 8.69 and 25.04 kD (**Table S4**), respectively. Accession number of the sequence was MK913362 in the GenBank database.

### Analysis of Phylogenetic Tree and Protein Sequence Alignments

**Figure 2:**
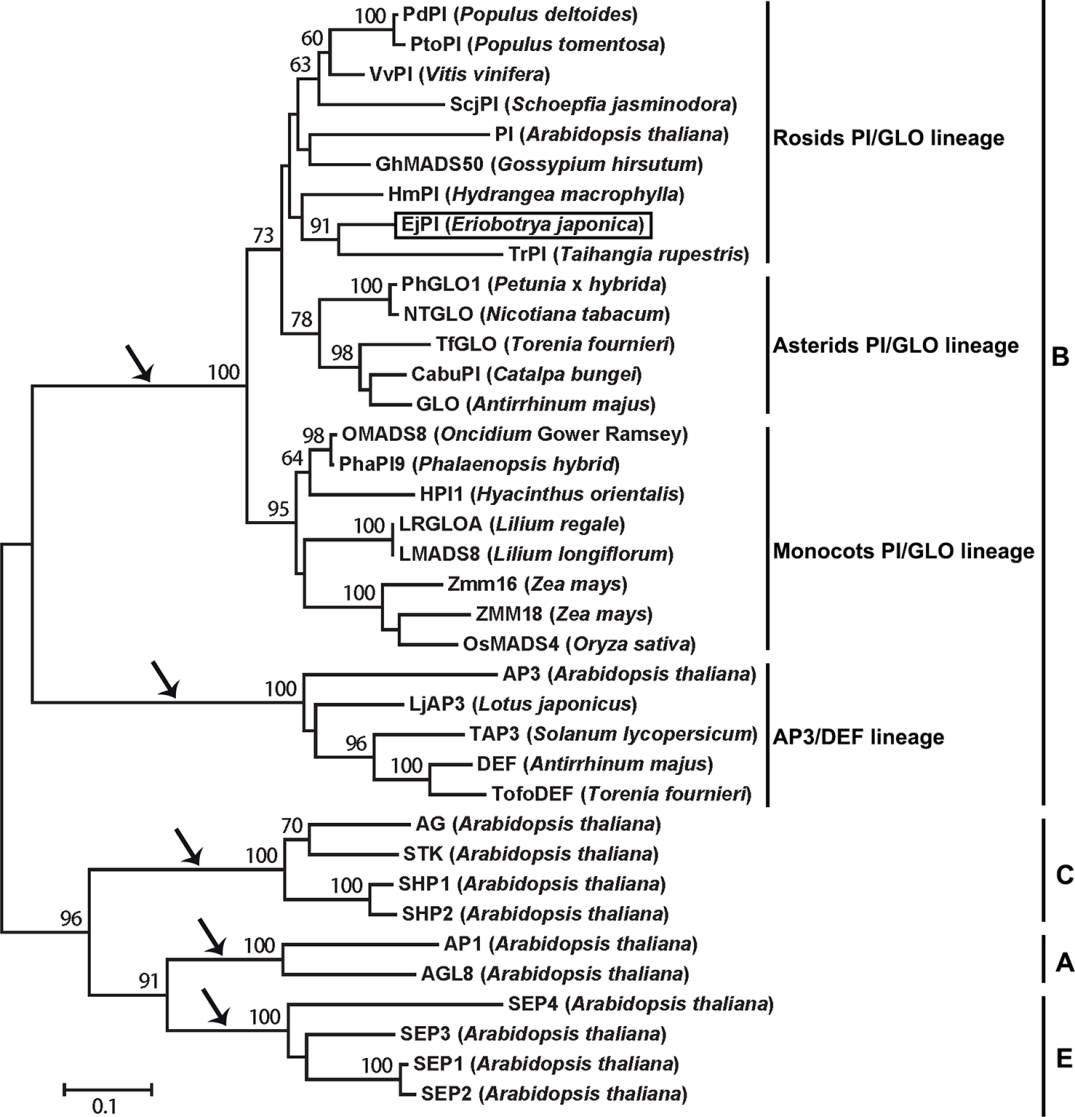
Phylogenetic analysis of PI/GLO-like MADS-box proteins. The EjPI protein sequence is blasted with twenty-six B-class proteins from other angiosperms, with two A-class proteins, four C-class proteins and four E-class proteins as out group. Black arrows show that the gene lineages are obtained through gene duplication. EjPI protein is marked. PI, *PISTILLATA*.

**Figure 3:**
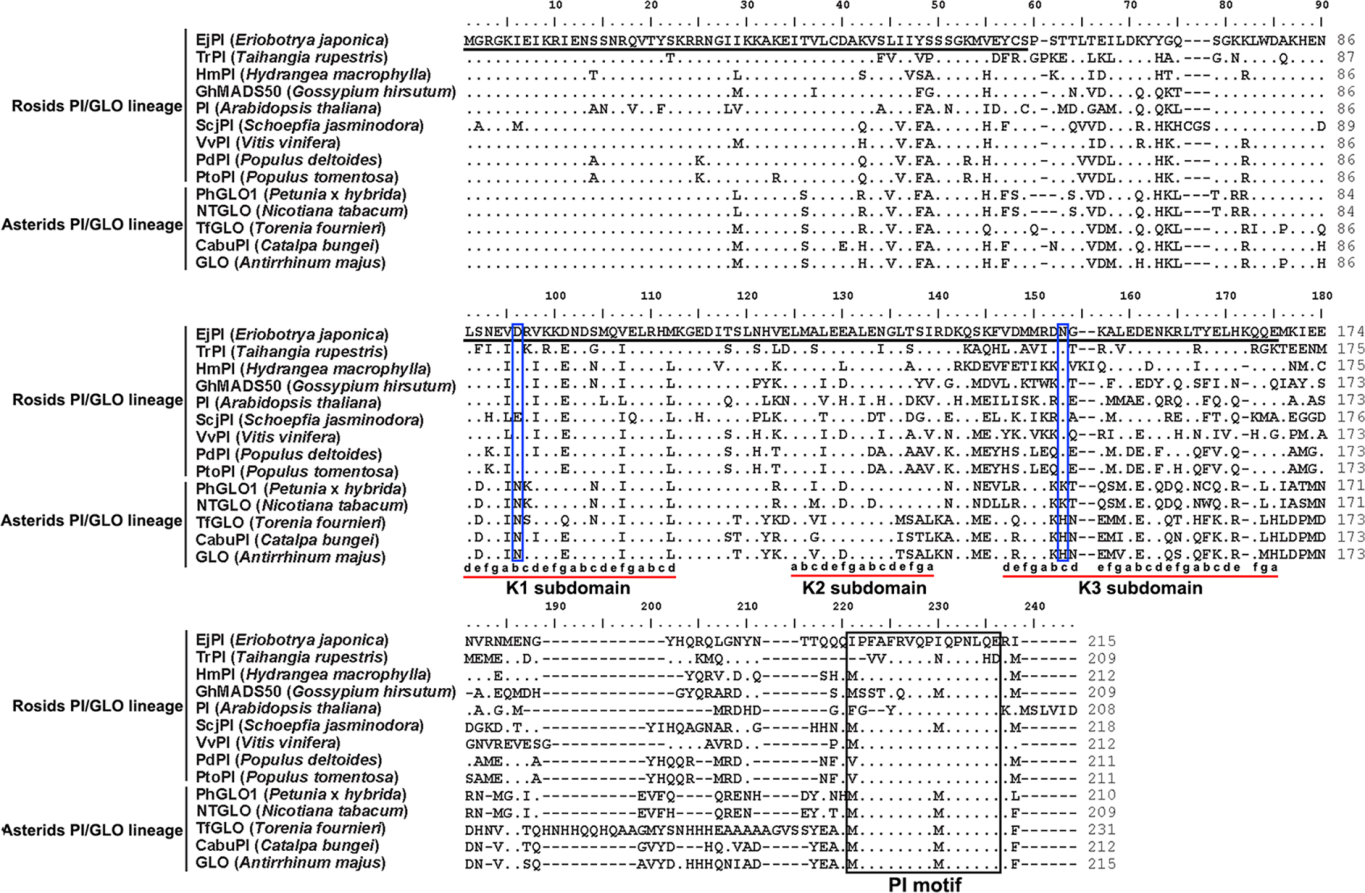
Sequence comparisons of EjPI and the other PI/GLO orthologous proteins. First underlined region represents the MADS domain. Second underlined region represents the K domain. The PI-motif is boxed. Dots indicate the amino acid residues identical to EjPI. Dashes are introduced into the sequences to improve the alignment. The K domain contains K1, K2, and K3 subdomains with (abcdefg)n heptad repeats (Yang et al., 2003), which are also underlined. Meanwhile, Asp-92/Glu-92 and Asn-149 in K1 and K3 subdomains in rosids PI/GLO lineage are boxed.

Analysis of phylogenetic tree showed that the *EjPI* gene is assigned to the rosids PI/GLO lineage (**Figure 2**). Conceptual translation reveals that *EjPI* encode 215 amino acids (aa) including a 59-aa M domain, a 27-aa I domain, an 82-aa K domain, and a 47-aa C domain from N- to C-terminus (**Figure 3**). Among these domains, the M, I, and K domains were conserved in these aligned PI orthologous proteins. The K domain contains K1 (87-108), K2 (121-135), and K3 (143-169) subdomains with (abcdefg)n heptad repeats, which could potentially mediate protein interaction (Yang et al., 2003; Yang and Jack, 2004). In comparison to K domain of asterids PI/GLO lineage, K1 subdomain contains substitution of polar neutral amino acid (Asn-92) to acidic amino acid (Asp-92/Glu-92), and K3 subdomain contains substitution of basic amino acid (Lys-149/His-149) to polar neutral amino acid (Asn-149) in rosids PI/GLO lineage, respectively. However, the C domain, which includes a distinctive PI motif, showed more variable than the other domains (**Figure 3**).

### Subcellular Localization and Spatial Expression of *EjPI*

**Figure 4:**
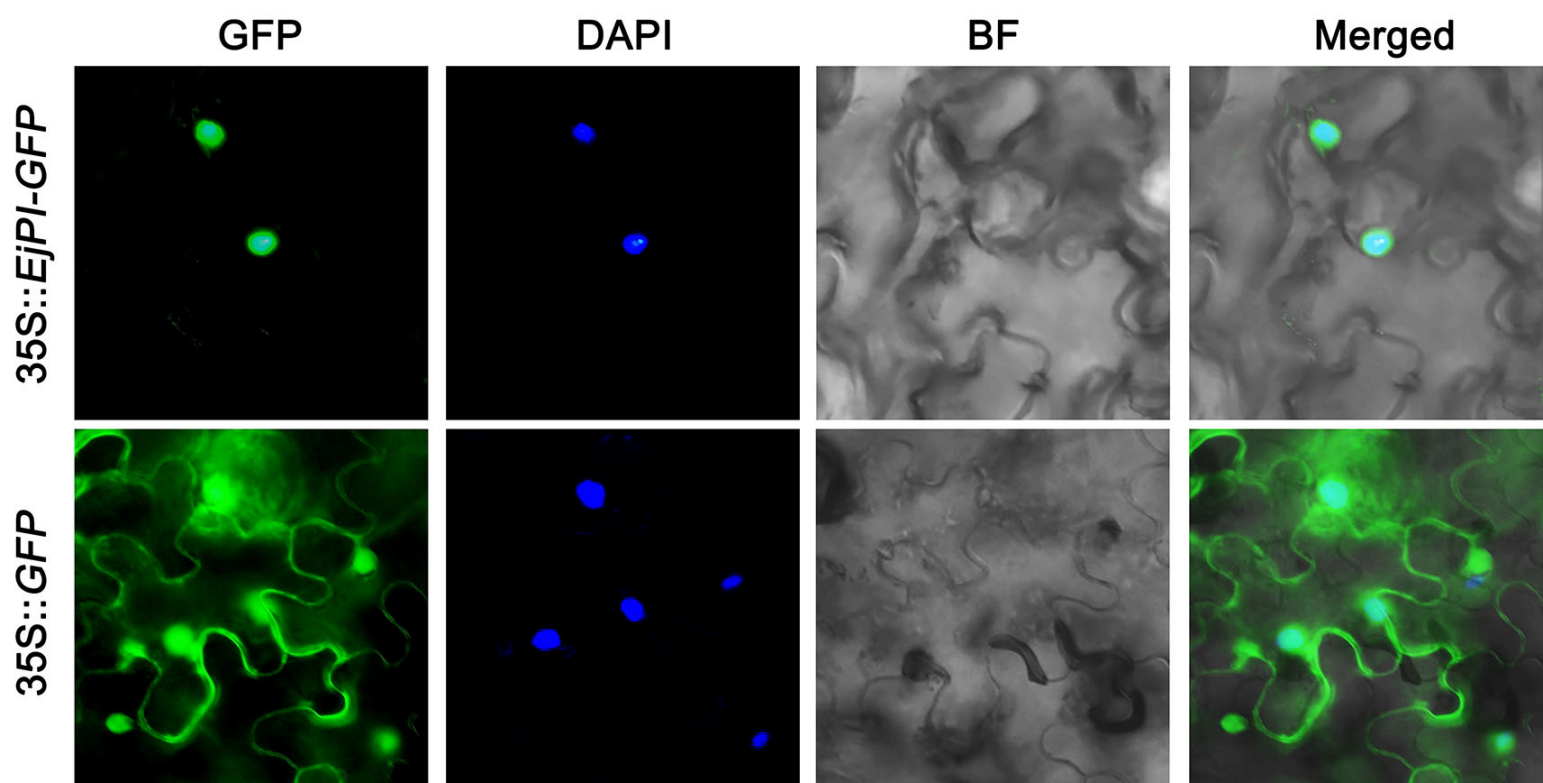
Subcellular localization of *EjPI*. GFP, GFP fluorescence; 4,6-diamidino-2-phenylindole (DAPI) staining shows nuclear localization; BF, bright-field; Merged, merged image of GFP and DAPI. GFP, green fluorescent protein.

To observe the subcellular localization of *EjPI*, EjPI-GFP fusion proteins transiently expressed in leaf epidermal cells in *N. benthamiana*. Fluorescence from 35S::GFP was as control and detected in both the nucleus and cytoplasm, but the fluorescence from 35S::EjPI-GFP was detected only in the nucleus (**Figure 4**).

**Figure 5:**
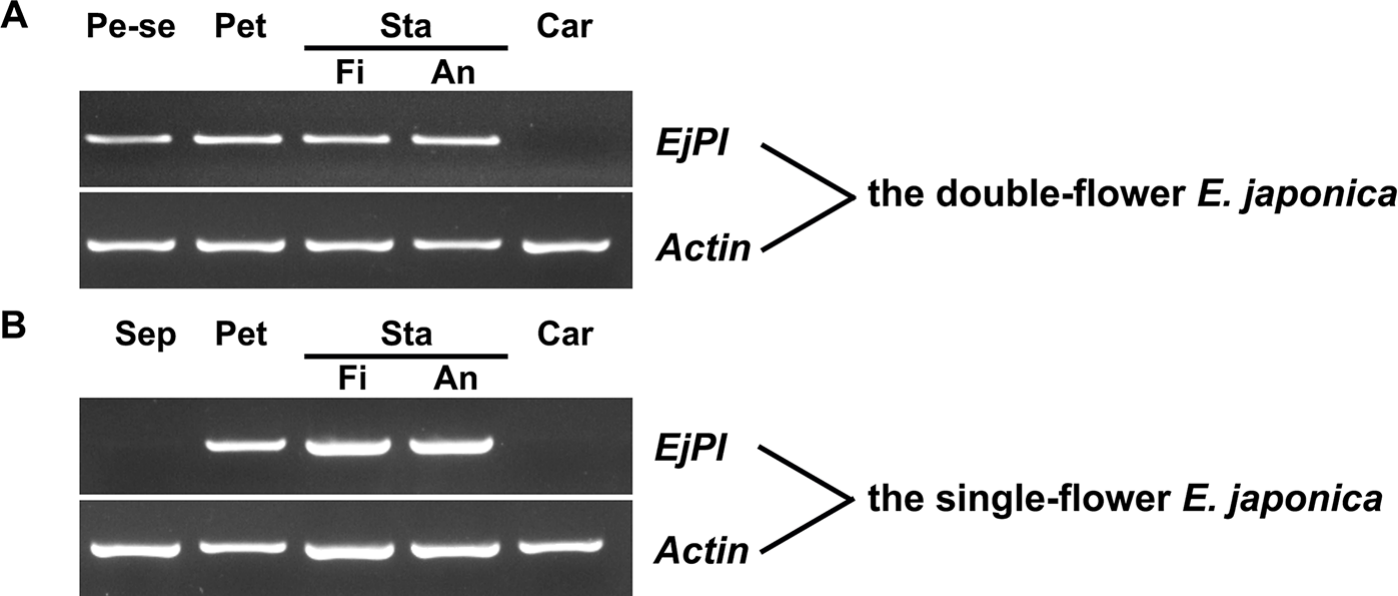
Spatial expression of *EjPI* in single-flower and double-flower *E. japonica* by semi-quantitative RT-PCR. **(A)** Spatial expression of *EjPI* in double-flower *E. japonica*. **(B)** Spatial expression of *EjPI* in single-flower *E. japonica*. Sep, sepals; Pe-se, petaloid sepals; Pet, petals; Sta, stamens; Car, carpels; Fi, filaments; An, anthers.

To analyze spatial expression pattern of *EjPI*, the semi-quantitative RT-PCR were performed in single-flower and double-flower *E. japonica*. In flower buds of single-flower *E. japonica*, *EjPI* was transcribed only in the petals, filaments and anther, but not in sepals and carpels (**Figure 5**). However, in the flower buds of double-flower *E. japonica*, *EjPI* was transcribed in petaloid sepals, petals, filaments, and anthers (**Figure 5**).

**Figure 6:**
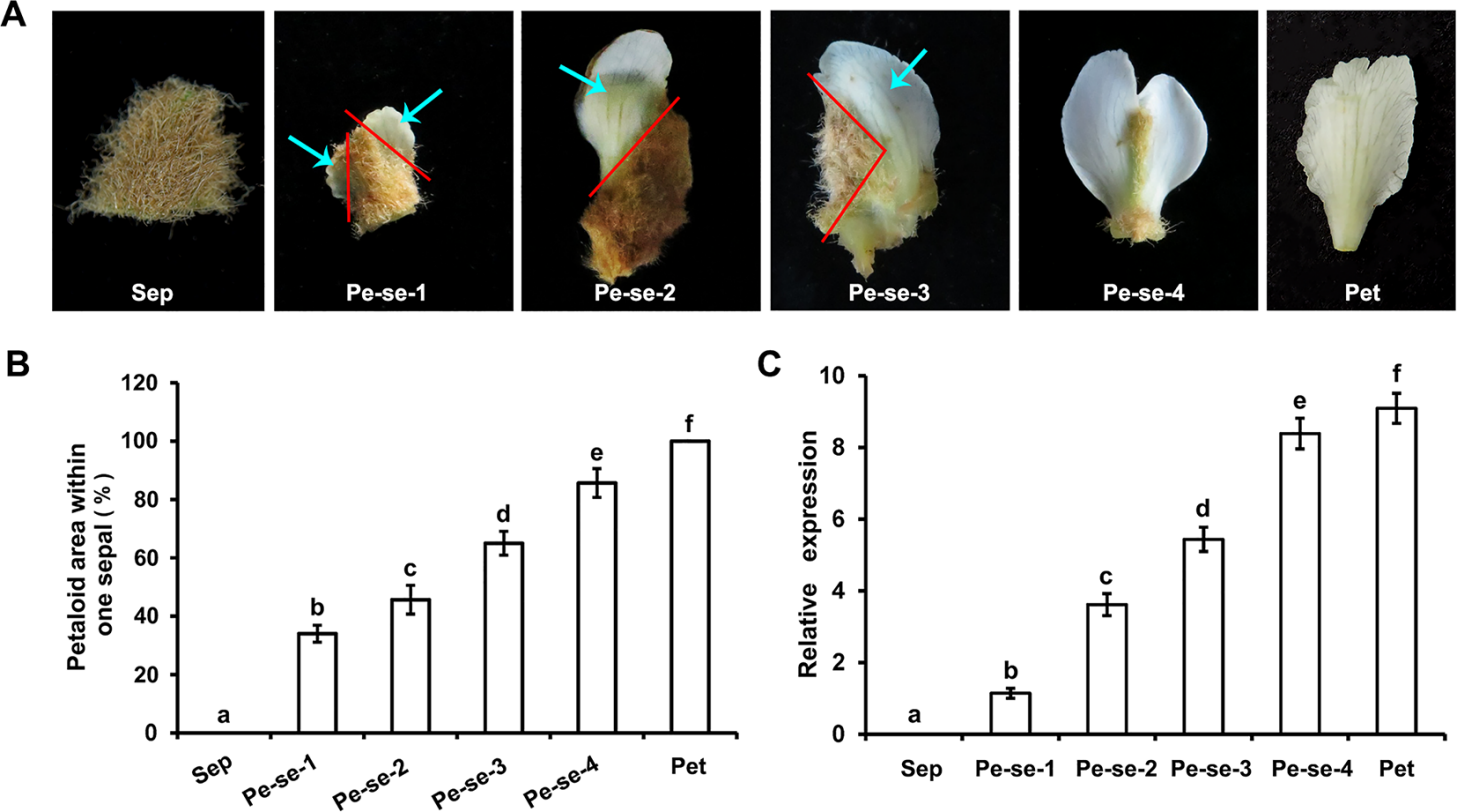
Relative expression levels of *EjPI* in petaloid sepals in double-flower *E. japonica* by qRT-PCR. **(A)** Different types of petaloid sepals. **(B)** Petaloid area within one sepal. **(C)** Relative expression levels of *EjPI* in petaloid sepals. Sep, sepals; Pe-se, petaloid sepals; Pet, petals. Error bars indicate the standard deviation of three biological replicates. Different letters indicate significant differences (P < 0.05).

We analyzed *EjPI* expression levels in petaloid sepals of different areas within the sepals. The expression level of *EjPI* in larger petaloid area within a sepal was significantly higher than that in small petaloid area (**Figure 6**). The correlationship between petaloid area and *EjPI* transcript level was further investigated. There were high correlation coefficients between *EjPI* transcript level and petaloid area (Pearson’s correlation coefficient of 0.977, **Table S5**), indicated that increased *EjPI* expression level causes the increased petaloid area within a sepal.

### Functional Analyses of *EjPI in* Transgenic Arabidopsis

**Figure 7:**
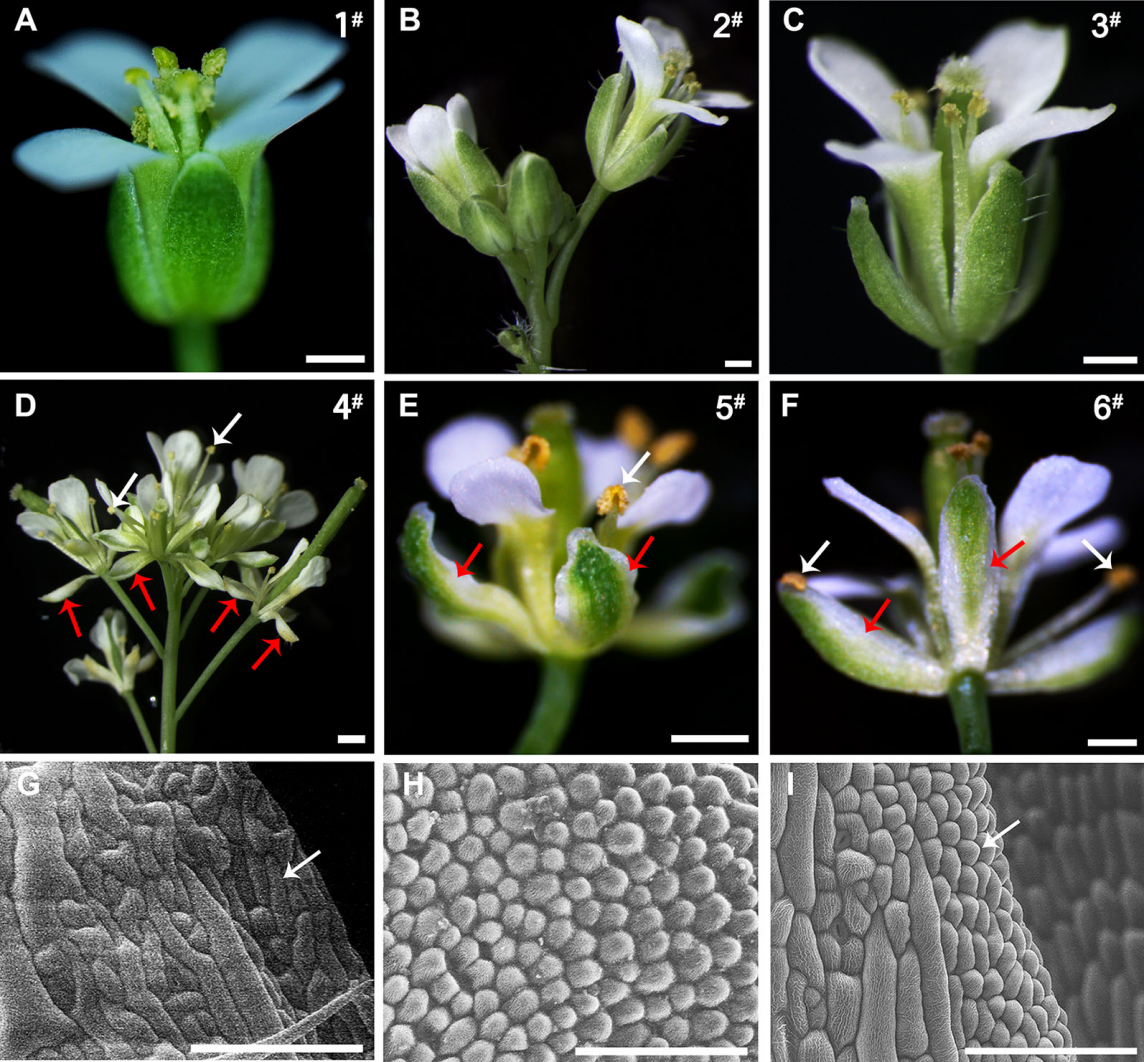
Comparison of the phenotypes of the wild-type and 35S::*EjPI* transgenic wild-type lines. **(A)** Flower of the wild-type Arabidopsis, showing tightly closed sepals and stamens (line 1^#^). **(B)** Inflorescence of the transgenic wild-type Arabidopsis with the pBI121 vector only (negative control, line 2^#^). **(C)** Flower of the transgenic wild-type Arabidopsis with the pBI121 vector only, showing no phenotypic alteration (line 3^#^). **(D)** Inflorescence of 35S::*EjPI*, showing completely separating petaloid sepals (red arrows) and opening stamens (white arrows) (line 4^#^). **(E)** Flower of 35S::*EjPI* transgenic wild-type Arabidopsis, showing green/white petaloid sepals in the first whorl (red arrows) (line 5^#^). **(F)** Flower of 35S::*EjPI* transgenic wild-type Arabidopsis, showing completely separating petaloid sepals (red arrows) and opening stamens (white arrows) (line 6^#^). **(G)** Cell shapes of adaxial surface in a wild-type Arabidopsis sepals, showing irregular cell margin; **(H)** Cell shapes of adaxial surface in petals in wild-type Arabidopsis; **(I)** Cell shapes of adaxial surface in petaloid sepals in 35S::*EjPI* transgenic wild-type lines, showing a petaloid margins (white arrow). Bars = 500 µm in **(A)**, **(B)**, **(C)**, **(D)**, **(E)**, and **(F)** and Bars = 100 µm in **(G)**, **(H)**, and **(I)**.

The *pi-1* Arabidopsis, which is a strong allele leading to a truncated protein product, results in the phenotype of full B-class loss-of-function. Flower of wild-type Arabidopsis has four sepals in the first whorl, four petals in the second whorl, six stamens in the third whorl and a carpel in the fourth whorl (**Figure 7A**). However, in homozygous *pi-1* mutant Arabidopsis, the second whorl petals are transformed into sepals and the third whorl stamens are replaced into a carpel (**Figure 9A**).

To analyze the functional characterization of *EjPI*, overexpression and complementation assays were conducted by ectopic expression of *EjPI* into wild-type and homozygous *pi-1* mutant Arabidopsis plants, respectively. We obtained thirty-one 35S::*EjPI* transgenic wild-type Arabidopsis lines. Among them, compared with untransformed wild-type plants, ten lines (32.26%) were phenotypically indistinguishable, but 21 remaining transgenic lines (67.74%) showed identical phenotypic alterations. In these transgenic lines with altered phenotypes, green/white petaloid sepals were produced in the first whorl (**Figures 7D–F**). Meanwhile, the first whorl sepals of Arabidopsis plants of wild-type and transgenic wild-type with the pBI121 vector were tightly closed even after pollination (**Figures 7A–C**). However, the first whorl petaloid sepals of 35S::*EjPI* transgenic wild-type lines opened immediately and separated completely after flower opening (**Figures 7D–F**).

Epidermis cellular shapes of adaxial side from the first whorl floral parts were further examined. Adaxial epidermis cellular shapes of sepal margin in a wild-type Arabidopsis were irregular (**Figure 7G**). Epidermis cellular shapes of the second whorl petals exhibited cone-shaped (**Figure 7H**). However, cell shapes of white portion of the petaloid sepals in transgenic wild-type Arabidopsis exhibited to be morphologically distinct from the first whorl sepal epidermis in wild-type plants, but similar to the epidermal cells of the second whorl petals (**Figure 7I**). Furthermore, the expression levels of *EjPI* in transgenic wild-type lines were confirmed by qRT-PCR (**Figure S3**).

**Figure 8:**
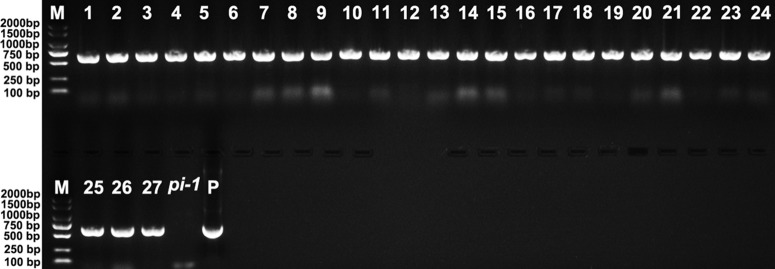
PCR analysis of 35S::*EjPI* transgenic homozygous *pi-1* mutant lines. Lane M: DL2000 DNA marker. Lane 1-27: the PCR with DNA of 35S::*EjPI* transgenic homozygous *pi-1* Arabidopsis as templates. Lane *pi-1*: the PCR with DNA of *pi-1* line containing the pBI121 vector only as a template. Lane P: the PCR with plasmid containing *EjPI* full-length CDS as a template.

**Figure 9:**
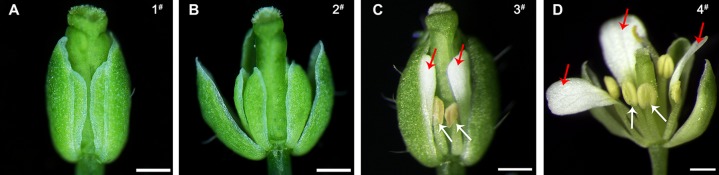
Phenotypic comparison of the homozygous *pi-1* mutant and 35S::*EjPI* transgenic homozygous *pi-1* Arabidopsis. **(A)** Flower of a homozygous *pi-1* mutant Arabidopsis (line 1^#^). **(B)** Flower of the transgenic homozygous *pi-1* mutant with the pBI121 vector only (negative control) (line 2^#^). **(C)** The flower of 35S::*EjPI* transgenic homozygous *pi-1* Arabidopsis, showing shortened petals (red arrows) and runtish stamens (white arrows) (line 3^#^). **(D)** Flower of 35S::*EjPI* transgenic homozygous *pi-1* Arabidopsis, showing normal petals (red arrows) and completely rescuing stamens (white arrows) (line 4^#^). Bars = 500 µm.

Twenty-seven 35S::*EjPI* transgenic homozygous *pi-1* mutant Arabidopsis lines were obtained by PCR analysis (**Figure 8**). Compared with untransformed homozygous *pi-1* lines, nineteen of these lines (70.37%) showed altered phenotypes in flower organ structure, but eight remaining transgenic plants (29.63%) were phenotypically indistinguishable. In these 35S::*EjPI* transgenic lines with altered phenotypes, eleven plants produced shortened petals in the second whorl and runtish stamens in the third whorl (**Figure 9C**). Eight transgenic homozygous *pi-1* lines produced normal petals in the second whorl and completely rescued stamens in the third whorl (**Figure 9D**). The expression levels of *EjPI* in transgenic lines were further confirmed by qRT-PCR. The Pearson correlation between *EjPI* expression level and phenotype alteration was significant in transgenic lines (*P*-value < 0.01) (**Table S6**). Meanwhile, *EjPI* expression levels of transgenic *pi-1* lines with normal petals and stamens were significantly higher than those of transgenic lines with runtish petals and stamens (**Figure S4**).

## Discussion

Characterization of key regulatory genes in model plants provides the opportunity to uncover the functional roles of orthologous genes in other plants. In model plants, such as Arabidopsis and Antirrhinum, *PI* homologues encode floral homeotic B-function MADS-box transcription factors and regulate petal and stamen identities (Trobner et al., 1992; Goto and Meyerowitz, 1994). In this study, we identified the sequence, phylogenetic evolution, and expression patterns of *EjPI* from single-flower and double-flower *E. japonica*. Meanwhile, ectopic expression of *EjPI* in Arabidopsis plants of wild-type and *pi-1* mutant was further conducted to confirm the functional roles in regulating floral organ identities.

Protein sequence and phylogenetic analysis showed EjPI protein was assigned to the rosids euPI lineage containing a highly-conserved M domain, an I domain, a less-conserved K domain, a highly divergent C-terminal domain and a distinctive PI motif at the C-terminal region. Therefore, *EjPI* encodes a typical class B-function MADS-box transcription factor according to the structural features of MADS-box proteins (Kim et al., 2004; Kaufmann et al., 2005). However, K1 and K3 subdomains both contain a single amino acid difference between rosids and asterids PI/GLO lineage. This difference may be related to the divergence of rosids and asterids in the core eudicot. Furthermore, the subcellular localization of EjPI was detected in the nucleus; this is consistent with the nuclear localization of *PI* in Arabidopsis (Mcgonigle et al., 1996).

In our study, *EjPI* was expressed in the petals, filament and anther in the flower buds of *E. japonica*. Spatial expression of *EjPI* matched well with that in some core eudicots, such as *Arabidopsis thaliana* (Goto and Meyerowitz, 1994), *Taihangia rupestris* (Lu et al., 2010), *Antirrhinum majus* (Trobner et al., 1992), *Torenia fournieri* (Sasaki et al., 2014), *Nicotiana tabacum* (Hansen et al., 1993), and *Catalpa bungei* (Jing et al., 2015). However, expression pattern of *EjPI* was different from that of the *PI* orthologs in some eudicots and monocots. In basal eudicot *Magnolia wufengensis*, *MawuPI* was mainly expressed in the first and second whorl inner tepals and the third whorl stamens (Liu et al., 2018). In *Fagopyrum esculentum*, *FaesPI* was expressed only in stamens (Fang et al., 2015). In monocot *Oncidium* Gower Ramsey, *OMADS8* expression was restricted in all floral organs, such as sepals, petals, lips, stamen, and carpel (Chang et al., 2010).

Beside expression in the petals, filaments and anthers, *EjPI* was also transcribed in petaloid sepals in double-flower *E. japonica*. Furthermore, there was a high correlation between the expression level of *EjPI* and petaloid area within a sepal in double-flowers *E. japonica*. Previously, compared with the sepal in *Papaver somniferum*, higher expression level of *PapsPI* was detected in the petaloid sepals (Singh et al., 2014). In the flowers of monocot such as Zingiberales, Commelinales, Alismatales, and Liliales, expanded expression of class B genes into the first floral whorl was correlated with the formation of petaloid organs (Kanno et al., 2007; Hsu et al., 2015; Kanno, 2015; Theißen et al., 2016). These results suggested that altering expression pattern of *PI* orthologous genes caused the transformation from sepal to petaloid organ in flowers. However, in some basal angiosperms, expression patterns of *PI* orthologues in petaloid organs are variability, which can be found in the relative expression level of *PI* vs *paleoAP3*. The expression pattern differences of *PI* orthologues in petaloid organs between *E. japonica* and basal angiosperms might be due to functional diversification within *PI* and *AP3* gene lineages during the course of angiosperm evolution.

Functional analysis suggested that ectopic expression of *EjPI* in transgenic wild-type Arabidopsis caused the first whorl sepals replaced into petaloid sepals. Similar phenotypes of transgenic wild-type Arabidopsis were observed in the ectopic expression of *PI* homologues from rosids species such as Arabidopsis (Krizek and Meyerowitz, 1996; Lamb and Irish, 2003), and asterids species such as *C. bungei* (Jing et al., 2015), *T. fournieri* (Sasaki et al., 2010) and monocots such as *Lilium longiflorum* (Chen et al., 2012), *Agapanthus praecox* (Nakamura et al., 2005) and *Cymbidium faberi* Rolfe (Fei and Liu, 2019). For instance, ectopic expression of *PI* caused the transformation of the first whorl sepals to petaloid organs in transgenic wild-type Arabidopsis (Lamb et al., 2003). Ectopic expression of *CabuPI* in transgenic wild-type Arabidopsis produced homeotic conversion of sepals into petaloid sepals in the first floral whorl (Jing et al., 2015). In *T. fournieri*, *TfGLO* expression in sepals exhibited a petaloid sepal phenotype (Sasaki et al., 2010; Sasaki et al., 2014).

Further functional complementation assay showed that *EjPI* could substitute the endogenous *PI* gene in *pi-1* mutant Arabidopsis and rescue the identity of petals and stamens. The phenotype differences of *EjPI* between transgenic wild-type and *pi-1* Arabidopsis may due to an effect of *pi* genotype. However, transgenic phenotypes of *EjPI* in *pi-1* Arabidopsis differed from those of *PI* orthologs from Magnoliaceae such as *MAwuPI* (Liu et al., 2018), and from monocots such as *LMADS8*/*9* of *L. longiflorum* (Chen et al., 2012) and *CyfaPI* of *C. faberi* (Fei and Liu, 2019). Ectopic expression of these monocots *PI* orthologs only partially rescued petal formation in *pi* mutant Arabidopsis.

## Conclusions

In our study, *EjPI* was isolated and its expression pattern and functional characterization were analyzed. The relative expression level of *EjPI* in larger petaloid area within a sepal was significantly higher than that in small petaloid area. Ectopic expression of *EjPI* in transgenic wild-type Arabidopsis caused a petaloid sepal phenotype in the first floral whorl. These data revealed that expression pattern and function of *EjPI* are associated with the formation of petaloid sepals in double-flower *E. japonica*. This improves our knowledge of *PI* orthologous genes in *E. japonica*, and provides the potential application of *EjPI* for biotechnical engineering to create petaloid sepals in angiosperm. Meanwhile, we did not find the difference of amino acid sequences of EjPI between the single-flower and double-flower *E. japonica* on distinct phenotype. Therefore, future work should compare the difference in *EjPI* promoter between the single-flower and double-flower *E. japonica*.

## Data Availability Statement

The datasets generated for this study can be found in the MK913362 in the GenBank database.

## Author Contributions

YX and MS conducted the experiments and drafted the manuscript. WC and RH carried out the analyses of qRT-PCR and endogenous hormones. DJ, DW, SW, QL, and HD contributed to the data analysis. QG and GL provided plant tissues, laboratory facilities, and project supervision. All authors approved the final draft of the manuscript.

## Funding

This work was financially supported by National Key Research and Development Program of China (No. 2019YFD1000200), Natural Science Foundation of China (No. 31800600), Fundamental Research Funds for the Central Universities (XDJK2019AA001 and XDJK2011D011), Key Projects of Chongqing Science and Technology Commission (cstc2018jscx-mszdX0054).

## Conflict of Interest

The authors declare that the research was conducted in the absence of any commercial or financial relationships that could be construed as a potential conflict of interest.
