# Supplementary material for: Expression Pattern and Functional Characterization of PISTILLATA Ortholog Associated With the Formation of Petaloid Sepals in Double-Flower Eriobotrya japonica (Rosaceae)
Source: Front Plant Sci. 2020 Jan 17;10:1685. doi: 10.3389/fpls.2019.01685 (PMC6978688; doi:10.3389/fpls.2019.01685)
Supplement: Supplementary file 1 [file DataSheet_2.doc]

SUPPLEMENTARY MATERIAL


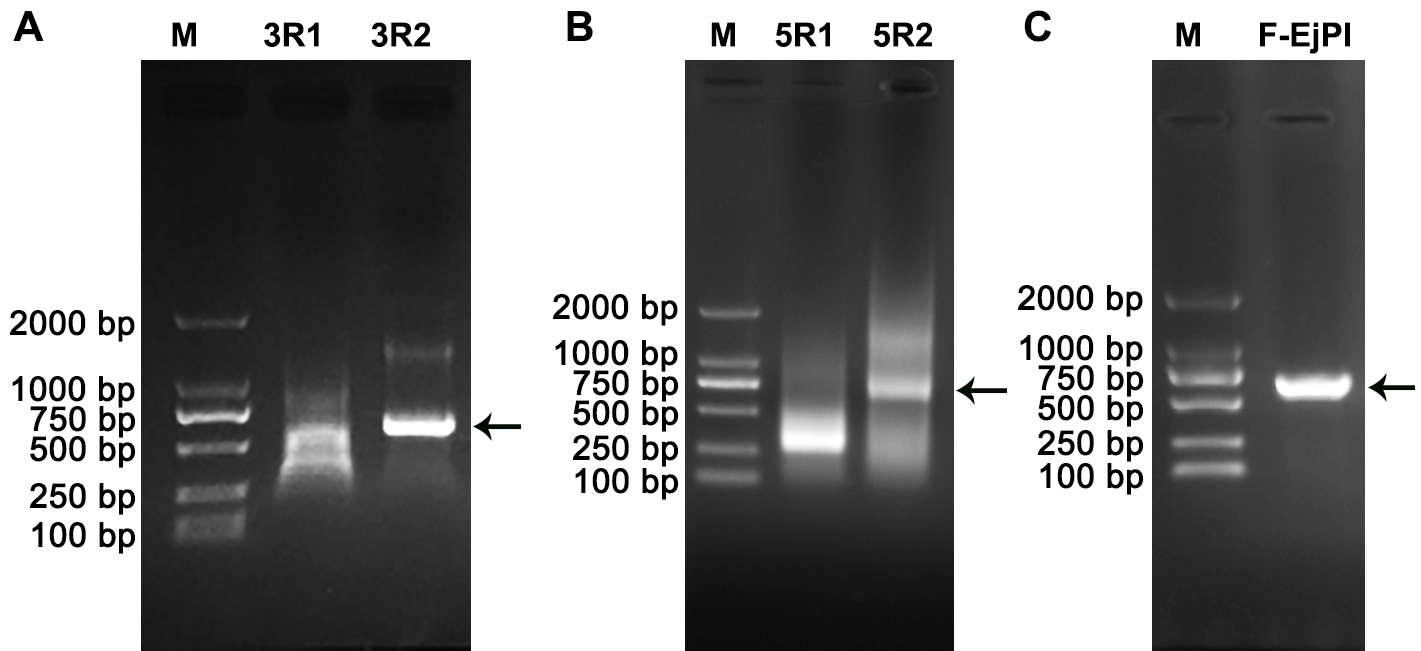


Figure S1 Isolation of *EjPI* from *E. japonica* flower bud. (A) 3′ RACE. (B) 5′ RACE. (C) Verification of the integrity of *EjPI*. The black arrows showed target gene fragments.


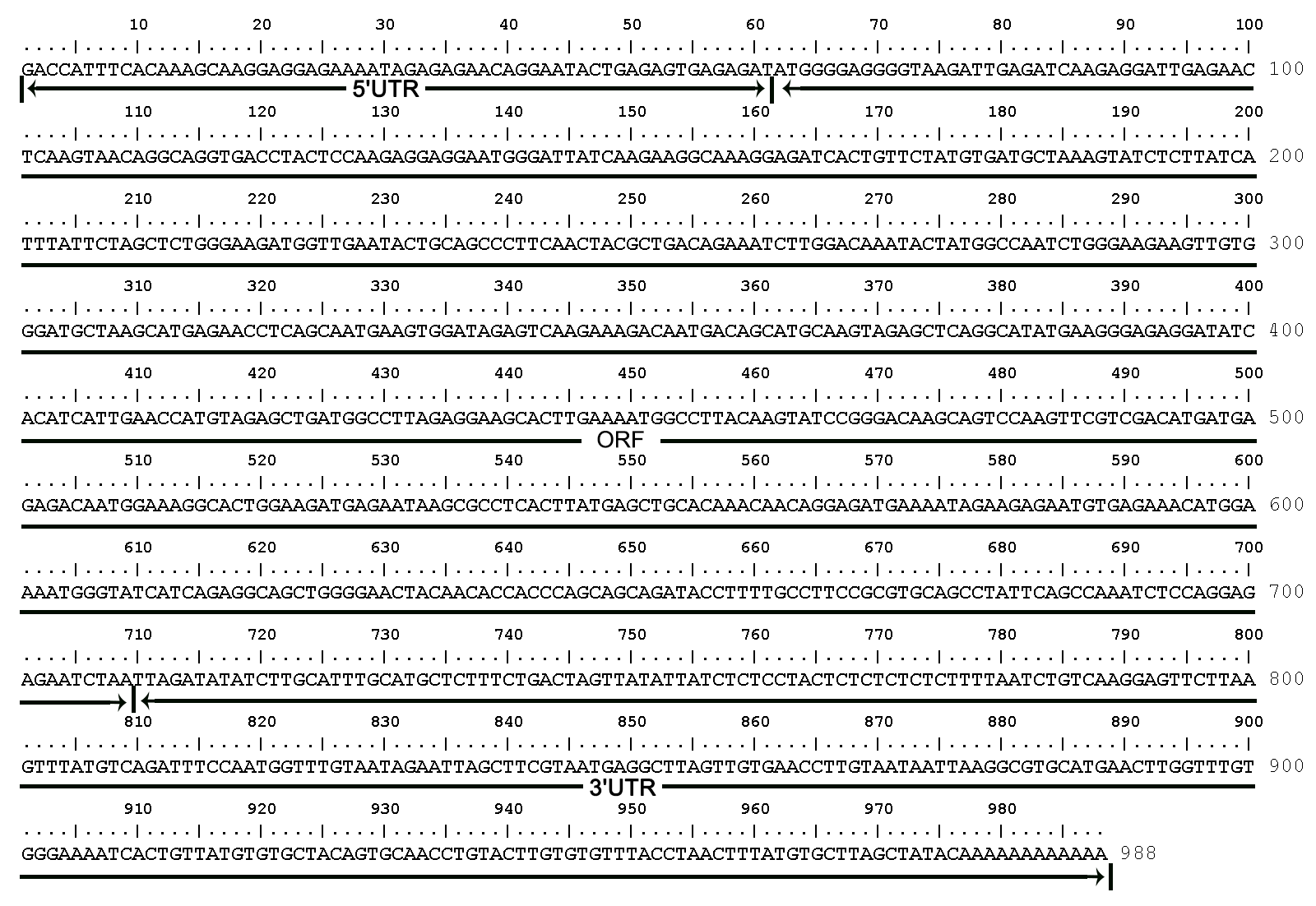


Figure S2 Sequences analysis of *EjPI* from *E. japonica*


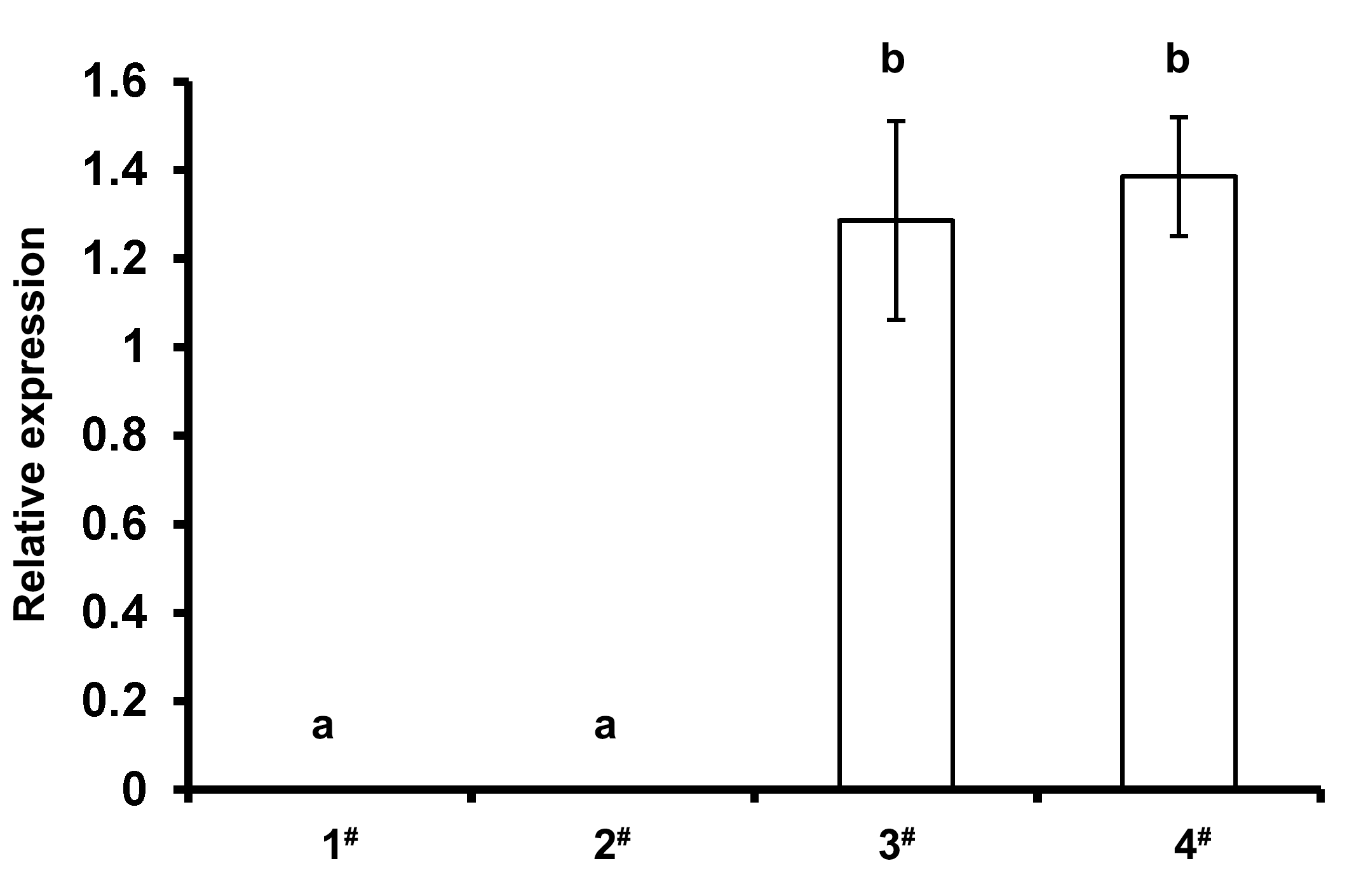


Figure S3 The expression levels of *EjPI* in transgenic wild-type lines. 1^#^: Wild-type *Arabidopsis*. 2^#^: The transgenic wild-type *Arabidopsis* with the pBI121 vector only (negative control). 3^#^ and 4^#^: The 35S::*EjPI* transgenic wild-type *Arabidopsis* with green/white petaloid sepals in the first whorl. The error bar indicates the standard deviation of three biological replicates. Different letters indicated significant differences (P < 0.05).


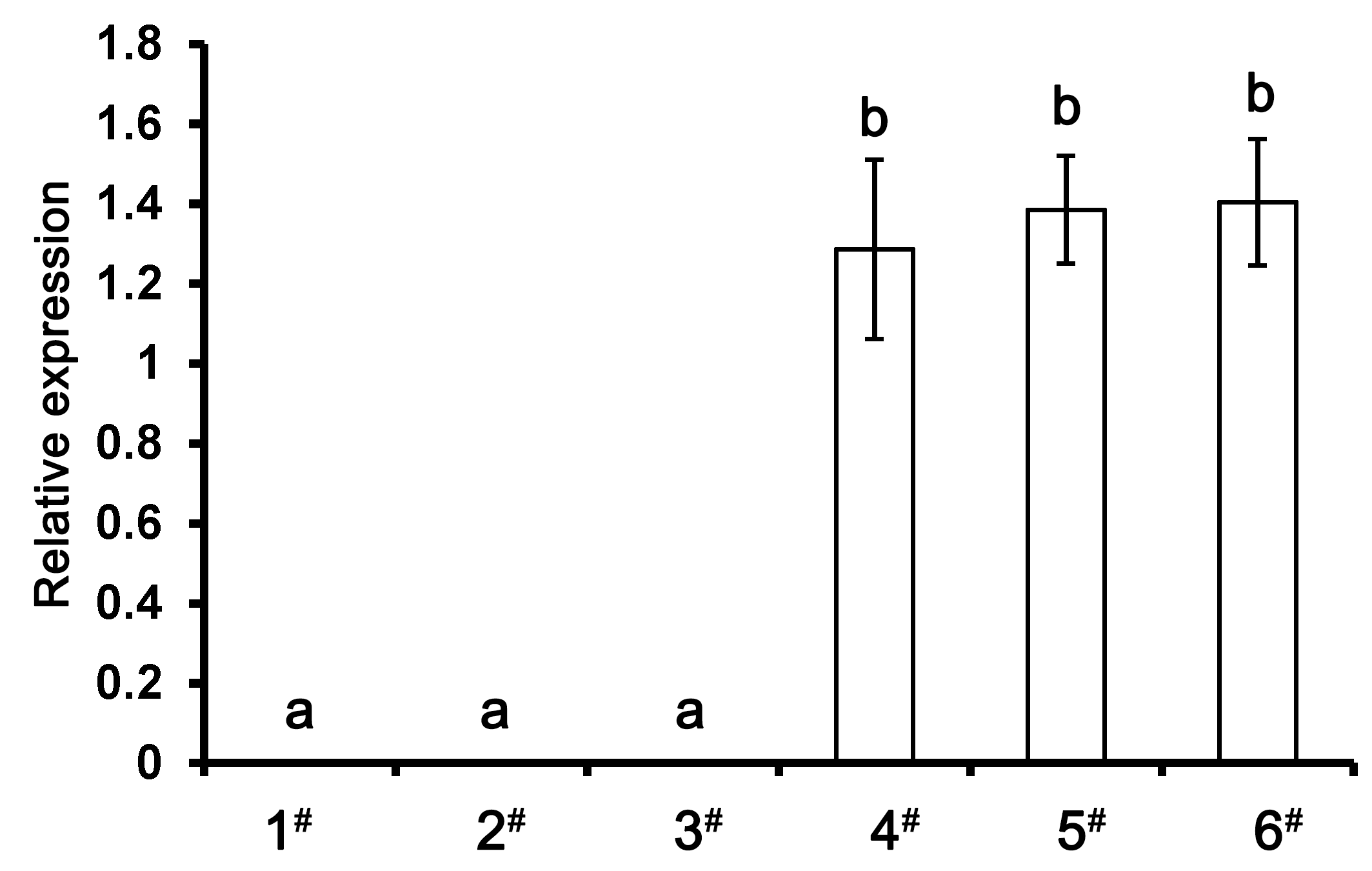


Figure S4 the expression levels of *EjPI* in different transgenic *Arabidopsis* lines. 1^#^: homozygous *pi-1* *Arabidopsis*. 2^#^: The transgenic homozygous *pi-1* mutant with the pBI121 vector only (negative control). 3^#^: The 35S::*EjPI* transgenic homozygous *pi-1* *Arabidopsis* with shortened petals and runtish stamens. 4^#^: The 35S::*EjPI* transgenic homozygous *pi-1* *Arabidopsis* with normal petals and completely rescuing stamens. The error bar indicates the standard deviation of three biological replicates. Different letters indicated significant differences (P < 0.05).

Table S1 Isolation of *EjPI* gene using the primer sequences and annealing temperatures of homology-based cloning and RACE.

| Primer | Primer sequences (5′ to 3′) | °C |
| --- | --- | --- |
| 3REjPI1 | ATCACTGTTCTATGTGATGCTA | 57 |
| 3′RACE Outer Primer | TACCGTCGTTCCACTAGTGATTT |
| 3REjPI2 | GTGGATAGAGTCAAGAAAGACA | 54 |
| 3′RACE Inner Primer | CGCGGATCCTCCACTAGTGATTTCACTATAGG |
| 5REjPI1 | AGATTTGGCTGAATAGGCTGCA | 56 |
| UPM Primer | TAATACGACTCACTATAGGGCAAGCAGTGGTATCAACGCAGAGT / CTAATACGACTCACTATAGGGC |
| 5REjPI2 | TATCTGCTGCTGGGTGGTGTTGTAG | 56 |
| UPM Primer | TAATACGACTCACTATAGGGCAAGCAGTGGTATCAACGCAGAGT / CTAATACGACTCACTATAGGGC |
| FLEjPIF | TAGAGAGAACAGGAATACTGAGAG | 57 |
| FLEjPIR | ATAATATAACTAGTCAGAAAGAGCA |

Table 2 The EjPI protein sequences, twenty-six B-class proteins from other angiosperms, with two A-class proteins, four C-class proteins and four E-class proteins were selected, including their family name and the Genbank accession numbers

| Protein | Species | [Family](app:ds:family) | accession number |
| --- | --- | --- | --- |
| PdPI | *Populus deltoides* | Salicaceae | ABS71831 |
| PtoPI | *Populus tomentosa* | Salicaceae | AGL09298 |
| VvPI | *Vitis vinifera* | Vitaceae | AFR53063 |
| ScjPI | *Schoepfia jasminodora* | Olacaceae | AFV74899 |
| PI | *Arabidopsis thaliana* | Brassicaceae | NM_122031 |
| GhMADS50 | *Gossypium hirsutum* | Malvaceae | AGW23352 |
| HmPI | *Hydrangea macrophylla* | Saxifragaceae | BAG68951 |
| TrPI | *Taihangia rupestris* | Rosaceae | CAB42988 |
| PhGLO1 | *Petunia × hybrid* | Solanaceae | AAS46018 |
| NTGLO | *Nicotiana t abacum* | Solanaceae | CAA48142 |
| TfGLO | *Torenia fournieri* | Scrophulariaceae | BAJ15423 |
| CabuPI | *Catalpa bungei* | Bignoniaceae | AJY60427 |
| GLO | *Antirrhinum majus* | Scrophulariaceae | CAA48725 |
| OMADS8 | *Oncidium* Gower Ramsey | Orchidaceae | HM140842 |
| PhaPI9 | *Phalaenopsis hybrid* | Orchidaceae | AAV28175 |
| HPI1 | *Hyacinthus orientalis* | Hyacinthaceae | AF134114 |
| LFGLOA | *Lilium regale* | Liliaceae | BAB91551 |
| LMADS8 | *Lilium longiflorum* | Liliaceae | AEI88009 |
| ZMM16 | *Zea mays* | Gramineae | AJ292959 |
| ZMM18 | *Zea mays* | Gramineae | CAC33849 |
| OsMADS4 | *Oryza sativa* | Gramineae | NM_001062125 |
| AP3 | *Arabidopsis thaliana* | Brassicaceae | NP_191002 |
| LjAP3 | *Lotus japonicus* | Leguminosae | AAX13301 |
| TAP3 | *Solanum lycopersicum* | Solanaceae | ABG73412 |
| DEF | *Antirrhinum majus* | Scrophulariaceae | CAA44629 |
| TofoDEF | *Torenia fournieri* | Scrophulariaceae | BAG24492 |
| AG | *Arabidopsis thaliana* | Brassicaceae | NP_567569 |
| STK | *Arabidopsis thaliana* | Brassicaceae | NP_001078364 |
| SHP1 | *Arabidopsis thaliana* | Brassicaceae | NP_001078311 |
| SHP2 | *Arabidopsis thaliana* | Brassicaceae | NP_850377 |
| AP1 | *Arabidopsis thaliana* | Brassicaceae | NP_177074 |
| AGL8 | *Arabidopsis thaliana* | Brassicaceae | NP_568929 |
| SEP1 | *Arabidopsis thaliana* | Brassicaceae | NP_001119230 |
| SEP2 | *Arabidopsis thaliana* | Brassicaceae | NP_186880 |
| SEP3 | *Arabidopsis thaliana* | Brassicaceae | NP_850953 |
| SEP4 | *Arabidopsis thaliana* | Brassicaceae | NP_849930 |

Table 3 The primer sequences of semi RT-PCR and qRT-PCR of *EjPI* gene

| Primer | Primer sequences (5′ to 3′) | °C |
| --- | --- | --- |
| RTEjPIF | TGCTAAGCATGAGAACCTCAGCAATGA | 58 |
| RTEjPIR | CAGCTGCCTCTGATGATACCCAT |
| RTEjactinF | AATGGAACTGGAATGGTCAAGGC | 54 |
| RTEjactinR | TGCCAGATCTTCTCCATGTCATCCCA |
| QEjPIF | TCTGGGAAGATGGTTGAATAC | 54 |
| QEjPIR | TGAGCTCTACTTGCATGCTGT |
| qEjactinF | AATGGAACTGGAATGGTCAAGGC | 54 |
| qEjactinR | TGCCAGATCTTCTCCATGTCATCCCA |
| qApiF | TCGACAAAGTCCGAGACCAC | 54 |
| qApiR | TCAATCGATGACCAAAGACA |
| qAactinF | CGTATGAGCAAGGAGTACAC | 54 |
| qAactinR | CACATCTGTTGGAAGGTGCT |
| PI-1MF | TACCAGAAGTTATCTGGCAAGAAATCATG | 54 |
| PI-1MR | TCTGATTCGCATAAGATTTGGTCT |

Table S4 The molecular weight and isoelectric points of EjPI protein

| Protein | Molecular weight | Theoretical isoelectric points |
| --- | --- | --- |
| EjPI | 25.04 kD | 8.69 |

Table S5 The correlation between the petaloid sepal area and the expression levels of *EjPI*

| Gene | Phenotype | correlation coefficient |
| --- | --- | --- |
| *EjPI* | Petaloid area | 0.977** |

** indicated significant differences (P < 0.01)

Table S6 The correlation between the expression levels of *EjPI* and the phenotype alteration in transgenic lines

| Transgenic lines | Relative expression level | Phenotype | Correlation coefficient |
| --- | --- | --- | --- |
| 1 | 1.00 ± 0.07 | 0 | 0.923** |
| 2 | 1.85 ± 0.40 | 0 |
| 3 | 0.70 ± 0.20 | 0 |
| 4 | 1.81 ± 0.45 | 0 |
| 5 | 1.76 ± 0.37 | 0 |
| 6 | 1.96 ± 0.20 | 0 |
| 7 | 2.26 ± 0.33 | 0 |
| 8 | 2.21 ± 0.31 | 0 |
| 9 | 9.25 ± 1.28 | 1 |
| 10 | 13.39 ± 1.87 | 1 |
| 11 | 9.47 ± 1.32 | 1 |
| 12 | 12.21 ± 1.69 | 1 |
| 13 | 9.44 ± 1.32 | 1 |
| 14 | 6.63 ± 2.75 | 1 |
| 15 | 9.04 ± 1.34 | 1 |
| 16 | 9.50 ± 1.68 | 1 |
| 17 | 11.64 ± 3.18 | 1 |
| 18 | 7.94 ± 1.66 | 1 |
| 19 | 10.35 ± 1.08 | 1 |
| 20 | 28.80 ± 5.09 | 2 |
| 21 | 36.82 ± 2.55 | 2 |
| 22 | 26.70 ± 2.87 | 2 |
| 23 | 25.49 ± 2.65 | 2 |
| 24 | 16.82 ± 1.75 | 2 |
| 25 | 19.32 ± 2.00 | 2 |
| 26 | 26.70 ± 2.87 | 2 |
| 27 | 22.19 ± 2.31 | 2 |

Transgenic lines with no phenotypic alterations are represented by 0. Transgenic lines with shortened petals and runtish stamens are represented by 1. Transgenic lines with normal petals and stamens are represented by 2. Mean ± SD, ** indicated significant differences (P < 0.01)
