# Supplementary figures and images for: Expression Pattern and Functional Characterization of PISTILLATA Ortholog Associated With the Formation of Petaloid Sepals in Double-Flower Eriobotrya japonica (Rosaceae)
Source: Front Plant Sci. 2020 Jan 17;10:1685. doi: 10.3389/fpls.2019.01685 (PMC6978688; doi:10.3389/fpls.2019.01685)

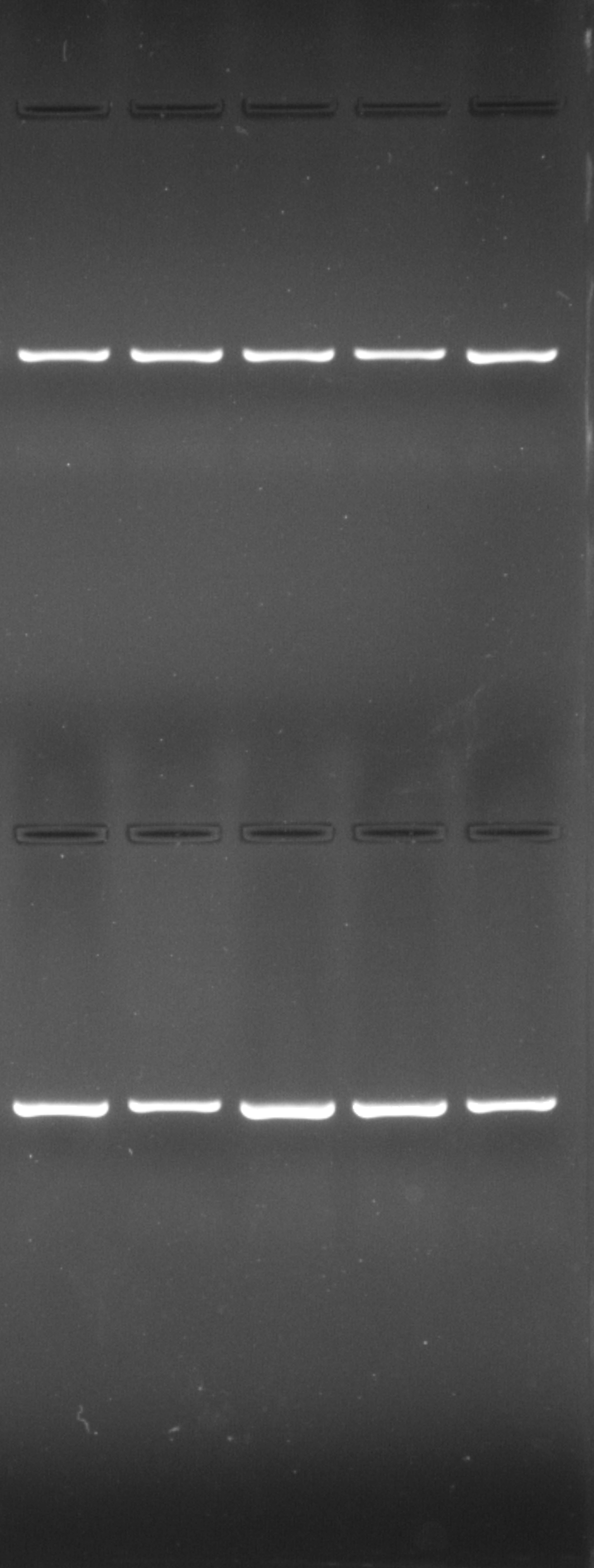

Supplement: Supplementary file 2 [file DataSheet_1.zip › 1 Actin of the double and single flower E. japonica in semi-quantitative RT-PCR.tif]

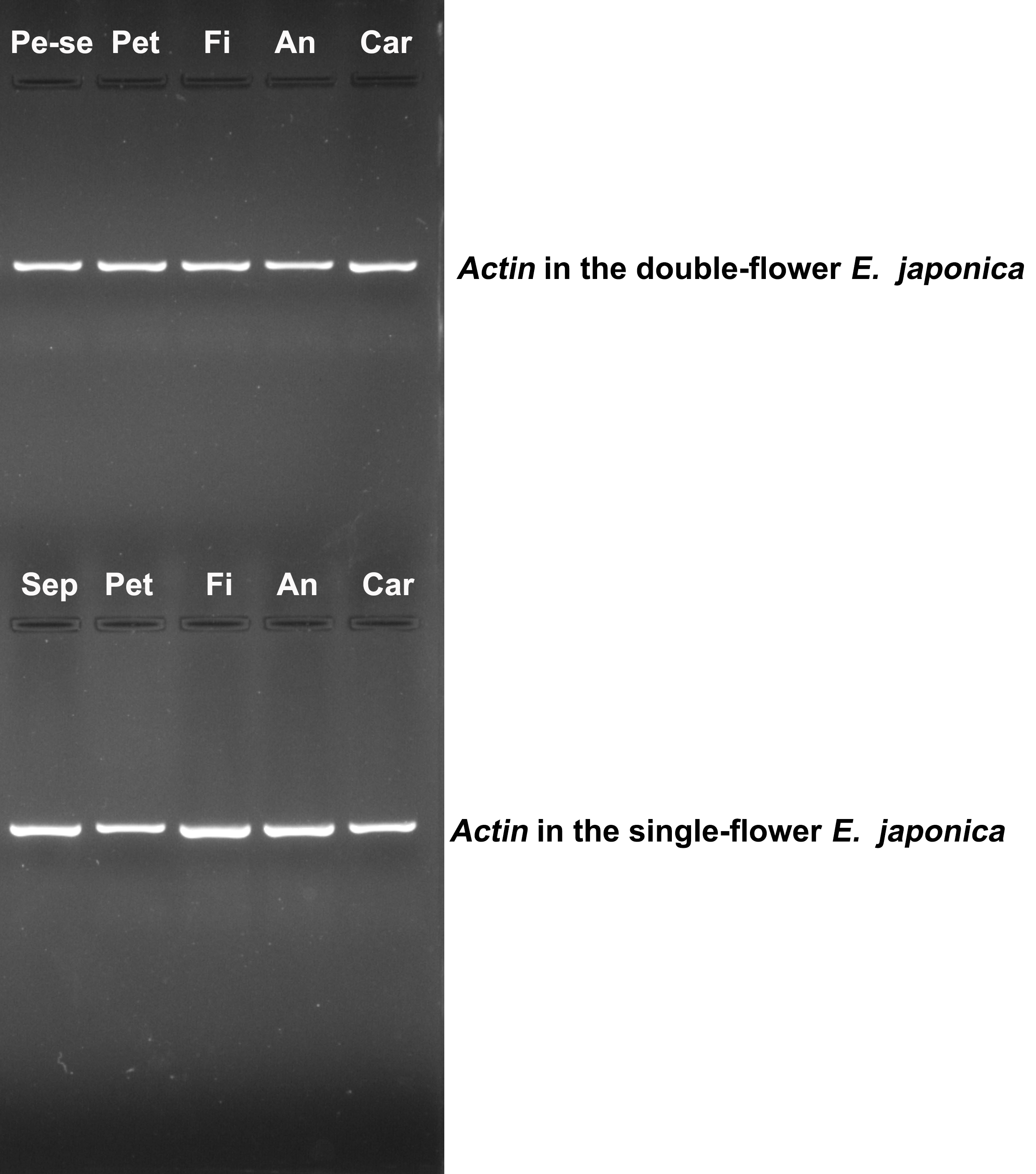

Supplement: Supplementary file 2 [file DataSheet_1.zip › 1-1 Caption Actin of the double and single flower E. japonica in semi-quantitative RT-PCR.tif]

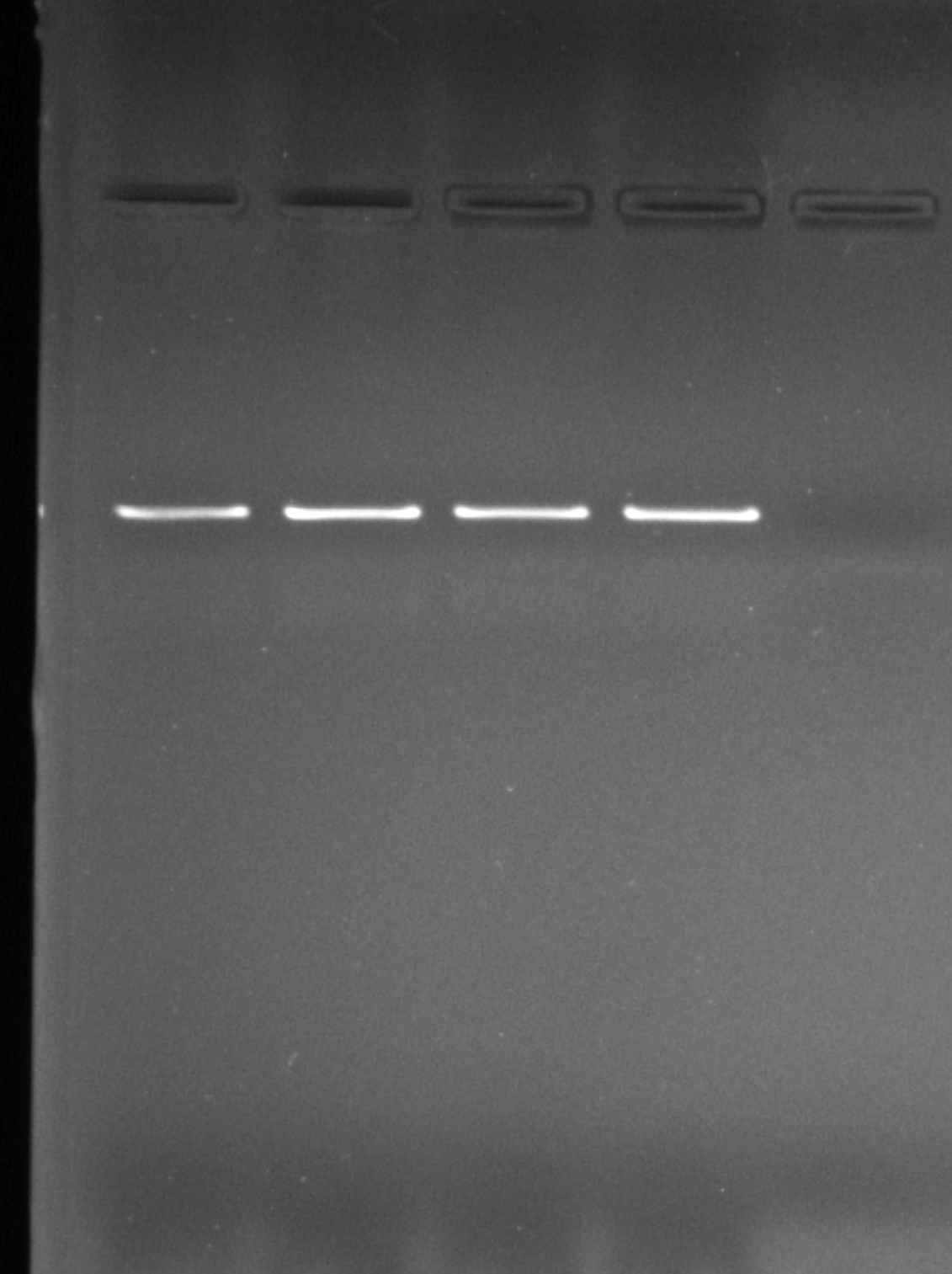

Supplement: Supplementary file 2 [file DataSheet_1.zip › 2 EjPI in double-flower E. japonica.tif]

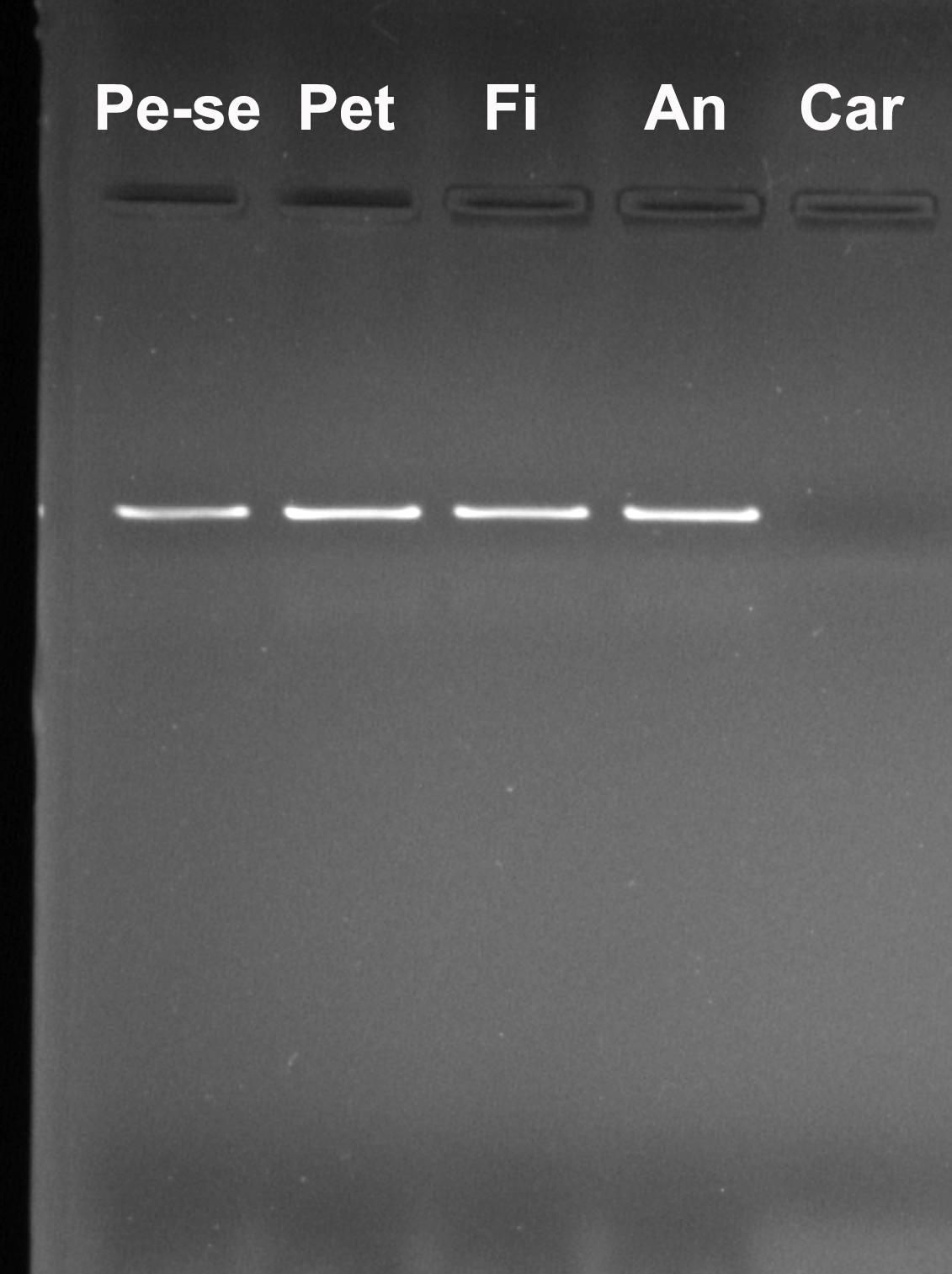

Supplement: Supplementary file 2 [file DataSheet_1.zip › 2-1 Caption EjPI in double-flower E. japonica.tif]

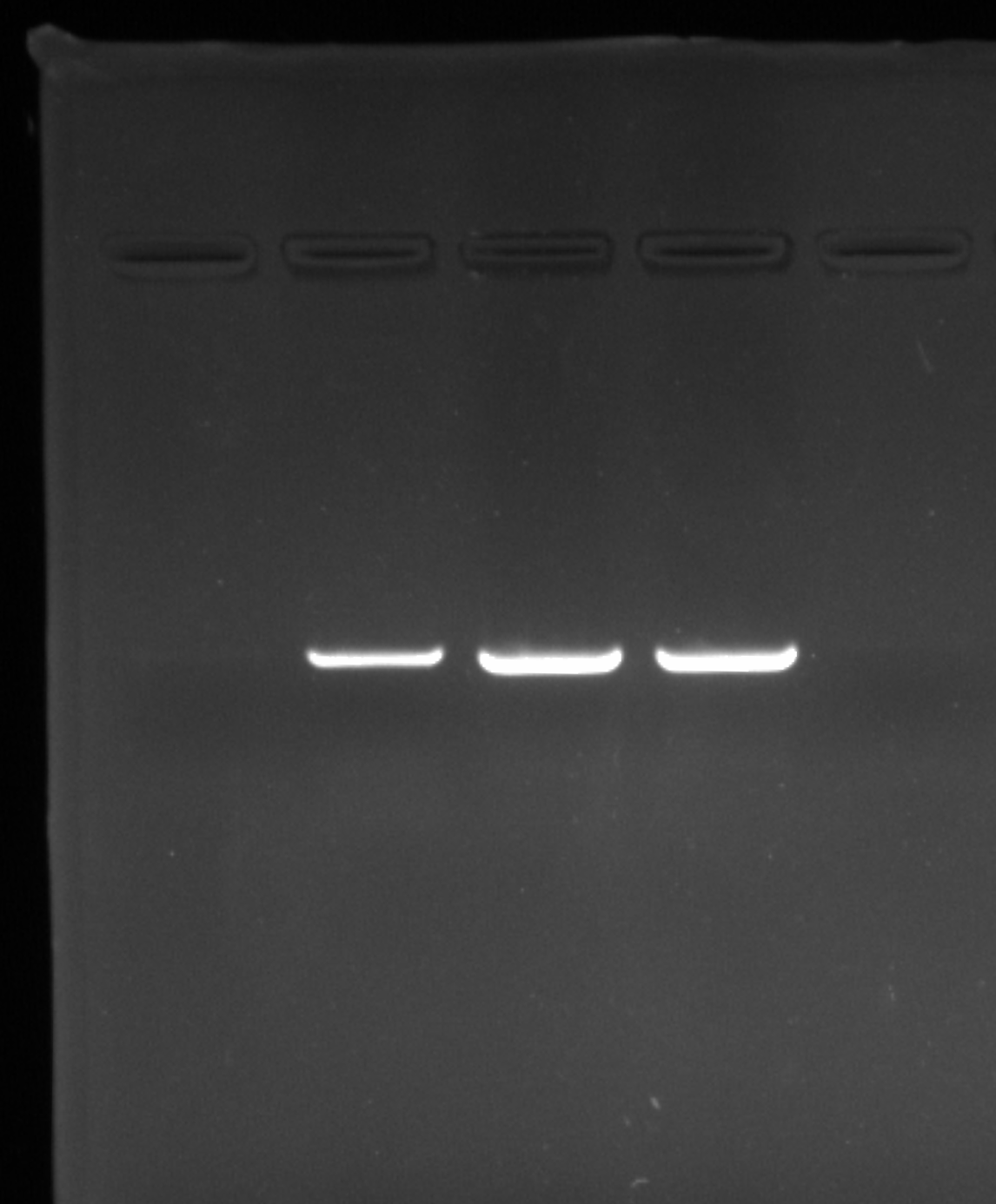

Supplement: Supplementary file 2 [file DataSheet_1.zip › 3 EjPI in single-flower E. japonica.tif]

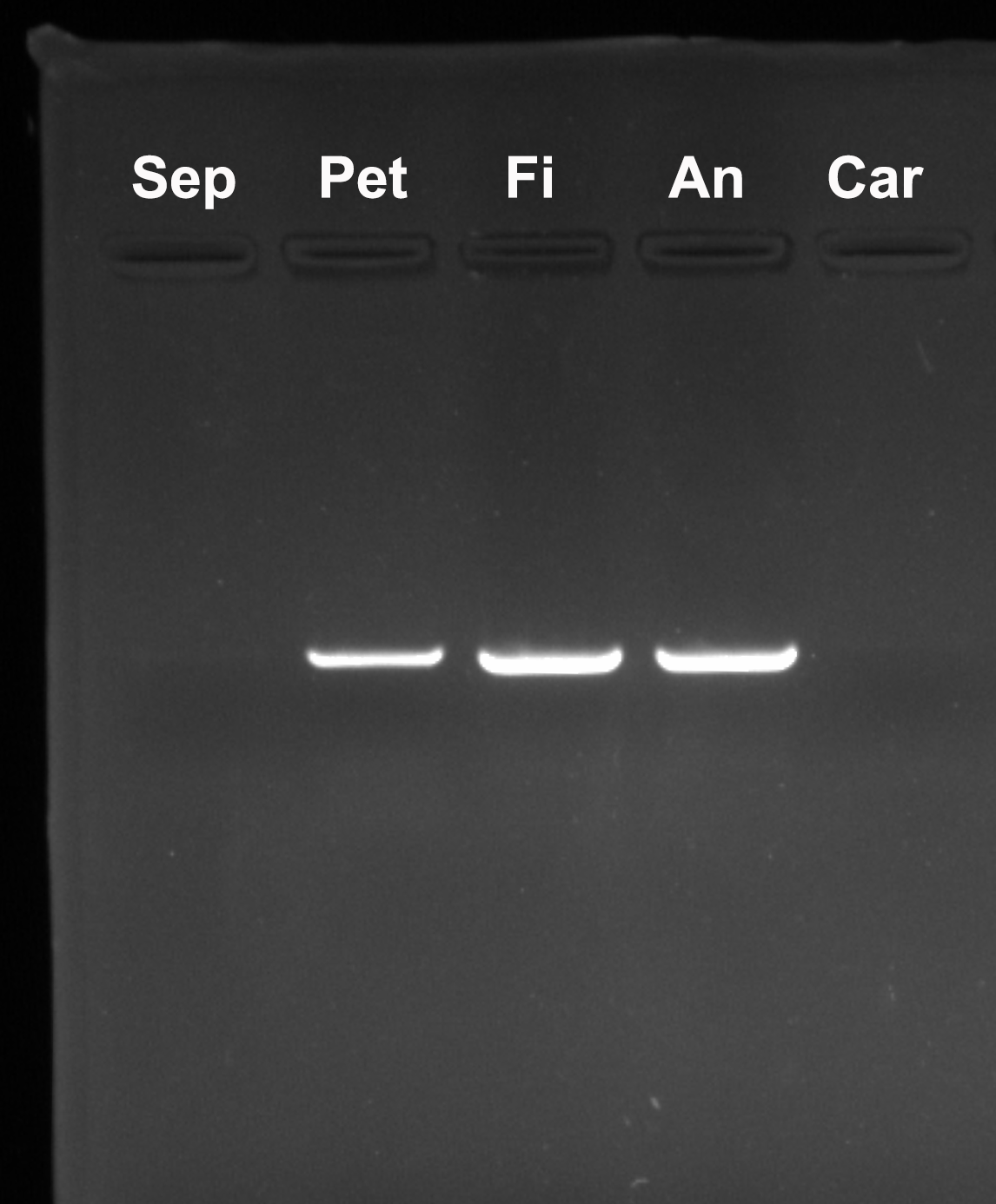

Supplement: Supplementary file 2 [file DataSheet_1.zip › 3-1 Caption EjPI in single-flower E. japonica.tif]
