# Supplementary material for: Expression Pattern and Functional Characterization of PISTILLATA Ortholog Associated With the Formation of Petaloid Sepals in Double-Flower Eriobotrya japonica (Rosaceae)
Source: Front Plant Sci. 2020 Jan 17;10:1685. doi: 10.3389/fpls.2019.01685 (PMC6978688; doi:10.3389/fpls.2019.01685)
Supplement: Supplementary file 2 [file DataSheet_1.zip › the image Captions for the blots.doc]

The image captions for the blots
